# A study on the interaction between logistics industry and manufacturing industry from the perspective of integration field

**DOI:** 10.1371/journal.pone.0264585

**Published:** 2022-03-03

**Authors:** Borui Yan, Qianli Dong, Qian Li, Lei Yang, Fahim U. I. Amin

**Affiliations:** 1 School of Economics and Management, Chang’an University, Xi’an, Shaanxi, China; 2 College of Transportation Engineering, Chang’an University, Xi’an, Shaanxi, China; University of Almeria, SPAIN

## Abstract

Studying the linkage between manufacturing industry and logistics industry is conducive to explore and improve the efficiency of the common development of them. In order to study the interaction of logistics industry on the development of manufacturing industry and the development of two-industry-linkage, it first calculates the high-quality development level of logistics industry and manufacturing industry, then uses the coupling coordination model to theoretically analyze and empirically test the coupling and coordinated development level of high-quality development of logistics industry and manufacturing industry from three aspects: coupling degree, coordination degree and coupling coordination degree, and based on the perspective of integration field theory, it takes the three basic synthetic fields of logistics integrator, logistics base-nuclear and logistics connection-key as the analysis dimension, PVAR model was introduced for in-depth analysis the impact of logistics industry on manufacturing industry and the level of the two-industry-linkage. It was found that the high-quality development of China’s logistics industry and manufacturing industry is close on the whole, and the development trend is consistent, the high-quality development of them is mainly caused by the change of scale, but there is no obvious change in technical efficiency, which also provides a way for the high-quality development of the two-industry-linkage in the future. The two-industry-linkage mostly belongs to the situation of low-level mutual restriction, which has not yet reached a high level of mutual promotion, resulting in the overall coupling coordination degree basically in a state of barely coordination. The development of logistics industry and manufacturing industry need to go through certain practice and running in, when there is an error matching between the two, the logistics industry will inhibit the two-industry-linkage. When the economy develops to a certain extent, the expansion of the logistics system scale to the level of the two-industry-linkage is not necessarily beneficial, blindly exceeding the demand for logistics investment will cause a waste of resources, which is not conducive to the high-quality development of the logistics industry and the coupling and coordinated development of the two industries. In the long run, the change of the logistics basic-nuclear capacity, the logistics integrator scale and logistics connection-key level will have a positive impact on the change of green total factor productivity in manufacturing industry.

## Introduction

Economic development needs the optimization of industrial structure, the upgrading and value of manufacturing industry. Manufacturing and logistics belong to the core supply category of market economy and are important components of the real economy. In the environment of fierce global competition, great innovation pressure and short response time to customer needs, enterprises are facing the problem of improving their logistics system. The increasing trend of logistics digitization and autonomy in Industry 4.0 is changing the existing logistics process [1, 2]. The manufacturing industry needs to be upgraded, not only to realize the benefits of commodity production, but also to upgrade and change the functions of planning, supply chain logistics and even product development [3]. The logistics industry and related industries interact with each other, focusing on the core interests of their respective industries and forming the relationship of industrial cooperation and interaction under the principle of complementarity. Studying the linkage between manufacturing and logistics is conducive to exploring and improving the efficiency of the common development of them, and promoting the optimization of regional economic structure and industrial upgrading.

The concept of the two-industry-linkage was first put forward at the First Joint Development Conference of China’s Manufacturing Industry and Logistics Industry in September 2007. It means that the logistics industry and manufacturing industry form a reasonable division of labor and cooperation system through strategic adjustment to realize complementary advantages and coordinated development, so as to optimize the industrial structure, enhance the industrial energy level and the industrial competitiveness [4]. The two-industry-linkage has attracted the attention of more and more scholars and government departments, and the empirical research on the interaction between the two industries has gradually increased. For example, Fan et al. [5] proposed that integrating innovative resources and promoting linkage development have a certain positive effect on improving the efficiency of logistics enterprises in urban agglomeration. Liu et al. [6] proposed that the interactive development of manufacturing industry and logistics industry affects the price level by improving product supply capacity and consumption capacity, reducing trade costs. Su et al. [7] proposed that the coupling of the logistics industry and manufacturing industry has a positive impact on the productivity of the manufacturing industry. Some scholars studied the symbiotic evolution relationship between industries and even industrial integration [8, 9], and some scholars used the system coupling theory to describe the process of industrial integration [10]. Especially with the concepts of Industry 4.0 and Made in China 2025 put forward, there are more and more studies on the integrated development of the two industries. At the same time, environmental awareness generally promotes the development of sustainable supply chain management. Manufacturing enterprises try to seek sustainable business strategies to deal with the pressure of the market on corporate social responsibility, and sustainable logistics service has become one of the most practical strategies [11]. In the context of high-quality development, how the logistics industry affects the manufacturing industry and the level of two-industry-linkage needs to be studied.

The rest of this paper is arranged as follows. The second section is the collation of relevant literature, the third section is the methodology, and the fourth section is the analysis results, mainly including the interactive impact of logistics industry and the comprehensive technical efficiency of manufacturing industry, the interactive impact of logistics industry and the change rate of green total factor productivity of manufacturing industry, and the impact of logistics industry on the level of two-industry-linkage, the last section is the conclusion and future research direction.

## Literature review

In recent years, economic globalization and manufacturing resource globalization, as two key factors, have prompted enterprises to change their business processes in order to survive in a competitive environment. Logistics is the key factor of modern networked manufacturing [12], effective logistics control is the main factor determining the competitiveness of manufacturing industry [13], logistics performance has become the key factor for the success of modern manufacturing enterprises [14, 15], logistics management has also become an important part of many manufacturers’ competitive strategies [16, 17]. Logistics can help manufacturing and distribution meet the increasingly stringent requirements of the global market [18], while manufacturing control more directly affects logistics objects [19], and logistics performance can be improved by taking advantage of the flexibility potential in manufacturing and assembly [20]. Logistics engineering is a broader part of manufacturing engineering, which can create the most competitive manufacturing process in the whole supply chain.

The early research on logistics and manufacturing mainly focused on the impact of logistics on the cost of manufacturing enterprises and the role of improving market response ability [21–23]. Some studies show that when the strategy and structure of manufacturing enterprises are consistent with the inherent advantages in enterprise logistics selection, the performance will be higher [24], and some studies explore the best combination of manufacturing and logistics services [25]. Some studies began to explore how to optimize the selected intertwined system of manufacturing and logistics, so as to achieve the expected level of manufacturing system [26–28]. Some studies have also begun to explore the mode of the two-industry-linkage. For example, Yang [29] proposed that the industrial linkage mode between the logistics industry and the manufacturing industry can be divided into six types according to the degree of logistics self-support and outsourcing: complete logistics self-support, partial outsourcing of logistics business, establishment of logistics professional companies, logistics strategic alliance, takeover of logistics system, and complete outsourcing of logistics business.

Some studies discussed the relationship between the two industries, mainly using Logistic model, DEA, grey correlation analysis, and composite system coordination model, coupled coordination model, input-output analysis, VAR model, PVAR model, etc. The measurement indicators of the level of two-industry-linkage mainly include: influence coefficient and induction coefficient, symbiosis degree and symbiosis coefficient, grey correlation degree, grey grid correlation degree, comprehensive validity of collaborative development (DEA), order degree and coordination degree, coupling coordination degree. This paper will use PVAR model [30] and the coupling coordination degree [31] to analysis the level of two-industry-linkage.

Manufacturing industry and logistics industry are highly related, deeply complementary, and interactive development has become the inevitable trend of their development [32], and will even develop into the integration of logistics chain and supply chain [33], it even affects the manufacturing industry and international logistics operation [34]. At the same time, as more and more manufacturing enterprises realize the importance of environment, they also begin to pay attention to the cost, carbon emission and energy consumption in the logistics process related to manufacturing enterprises [35, 36], and begin to explore how to improve the environmental performance of logistics process [37]. Through the implementation of green efforts in the logistics system, manufacturing enterprises can improve their efficiency, in addition to realizing the basic organizational objectives of manufacturing enterprises, they can also obtain some other long-term benefits [38], such as green supply chain management practices are much useful to improve environmental sustainability through a reduction in carbon emissions and PM2.5, it spur economic growth in terms of providing trade opportunities around the globe [39]. Although the green logistics related to manufacturing industry has received a certain degree of attention [40], it is still worth studying how the logistics industry will affect the manufacturing industry and the two-industry-linkage under the background of green development.

The research on the high-quality development of manufacturing industry was born with the research on the high-quality development of China’s economy. The connotation of high-quality development of manufacturing industry is closely related to evaluation, which mainly includes three views. The first view is that high-quality development of manufacturing industry mainly includes qualitative development (such as export technology complexity) and quantitative development (total output value of manufacturing industry) [41]; The second view is that it is mainly the adoption of advanced technology and the optimal allocation of resources [42]; The third view is that it should be considered from multiple dimensions, which has been recognized by many scholars [43]. The dimensions recognized by most scholars include economic benefits, structural optimization, innovative development, green development, opening up and social effects, which are similar to the five dimensions of high-quality economic development "innovation, coordination, green, opening and sharing", it is reflected from the side that most of these dimensions of high-quality development of manufacturing industry are derived from the connotation of high-quality economic development, but this approach also leads to a hidden danger, that is, some measurement index systems of high-quality development of manufacturing industry established on this basis overlap with the index system of high-quality economic development. For example, when investigating the dimension of opening up, some studies used the index of foreign capital dependence, which was widely used in the measurement system of high-quality economic development. Although the high-quality development of manufacturing industry is an important representation of high-quality economic development, there are still many differences between the two. Moreover, if many indicators coincide, it is doubtful whether it will lead to the error of the measurement results in the model with both high-quality manufacturing development and high-quality economic development. In fact, when investigating the high-quality development of manufacturing industry under the background of high-quality economic development, manufacturing total factor productivity can be used to measure the high-quality development level of manufacturing industry, which has also been recognized by many scholars [44]. The core of high-quality development of manufacturing industry is the improvement of manufacturing productivity (efficiency), which is the basis for sustainable economic development and the transformation of economic growth mode from extensive to intensive [45]. There are usually three methods to measure the efficiency of manufacturing industry, the first method is labor productivity (total industry output / total employment) [46], the second method is output rate, and the third method is to estimate the efficiency by using data envelopment analysis or stochastic frontier production function. The third method is the one used more, but scholars selected different input-output indicators. Investment indicators include: number of employees [47], and capital stock [47], number of business units [48], net value of fixed assets [49], total energy consumption [47, 50], etc.; Output indicators include industrial sales value [51], main business income [48], total output value [50], industrial added value [52], pollution emission [47, 49], etc.

High-quality development of logistics is an integral part of high-quality economic development [53]. It includes at least two meanings, first, the logistics industry has high development quality, which is reflected in high efficiency, high service level, strong endogenous power, sound industry, green environmental protection, etc., with the characteristics of "innovation, coordination, green, opening and sharing"; Second, the logistics industry can serve the social economy and people’s life with high-quality, strongly support the national economic development and meet the people’s growing needs for a better life.

As for the evaluation system of logistics high-quality development, some studies have considered the internal and external environment of the logistics industry for evaluation. For example, the index system established by Mu et al. [54] includes the economic environment of the logistics industry, the scale level of it, the input level of it, the output effect of it, etc.; The index system established by Cheng et al. [55] includes economic development level, logistics demand, logistics industry scale, informatization level and infrastructure construction; Li [56] proposed that the development quality of logistics industry can be measured from three aspects: development efficiency, development structure and development environment; Li et al. [57] established evaluation indicators including low-carbon logistics environment, low-carbon logistics strength, low-carbon logistics potential and low-carbon logistics level from a low-carbon perspective. It is more evaluated from the perspective of input-output. For example, Cao et al. [58] proposed that relevant indicators include input (capital input of logistics industry, labor input of logistics industry) and output (scale of logistics industry, quality of logistics industry); The index system established by Lu [59] includes input (labor, capital), output (added value, goods turnover); In the index system established by Li [60], inputs include capital input of logistics industry (fixed asset investment of logistics industry), labor input (the number of logistics industry employees), energy input (energy consumption of logistics industry), and outputs include expected output (output value of logistics industry) and unexpected output (CO_2_ emission of logistics industry).

**Fig 1:**
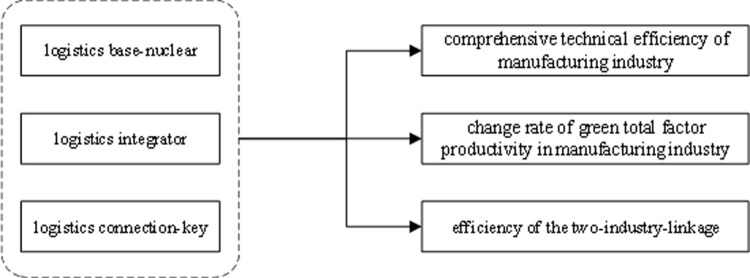
The logic framework diagram.

Generally, the impact of the logistics industry on the manufacturing industry and the two-industry-linkage only takes the efficiency of the logistics industry as the inspection index. However, the cooperation between logistics and manufacturing industry and even the two-industry-linkage are actually the integration and optimization process of logistics system and manufacturing system, the whole integration and optimization activities need to be considered. The integration field theory proposed by Professor Dong [61] regards all manual integration systems as integration fields, it holds that the integrated optimization is the general law of the artificial system, the integration field is the elaboration of the integrated optimization activities and general laws of the artificial integrated system, and it is the spatiotemporal distribution state of the synthetic field element under the action of the integrated force and integrated gravity in the field. The main basic units which is worth investigating separately in the integration field include integrator, base-nuclear, connection-key, field-line, field-boundary, etc., of which the first three basic units constitute the basic structure of the network chain [33]. The integrator is an active optimization adaptive integration subsystem, which dominates the integrated optimization process and has the nature of strategic subject, behavior subject and interest subject; The base-nuclear is the base carrying the field source, agglomeration and radiation field lines, and is an important node related to the integrated transfer connection and value gain; The connection-key constructs a stable structure of interaction, cooperation and coordination, and is the contact channel for the integrator and base-nuclear to gather and integrate resources; Field line is the track and performance of the composite action of multiple synthetic field elements dominated by the integrator. When using the integration field theory to analyze the development process of the two-industry-linkage, the integrator, base-nuclear (field source), connection-key and field line can be taken as the basic units. The integrator can be divided into manufacturing integrator and logistics integrator, they are usually the leading enterprises in the supply chain and logistics chain, they usually master the base-nuclear (field source), dominate the formation of supply chain or logistics chain, have strong integrated attraction and form joint forces, and affect or determine the field line performance; Base-nuclear is the carrier of field source, which can be divided into manufacturing base-nuclear and logistics base-nuclear, which mainly plays the role of attracting manufacturing integrator and logistics integrator; The connection-key can connect two or more synthetic field elements into a synthetic field, which usually include information type, resource type, function type, technology type, etc. it is the connection relationship between various business entities; In form, the field line is the integrated service track between various business entities, which can be reflected in business performance [4]. Therefore, we define the basic unit of the logistics system as the logistics integrator, logistics base-nuclear and logistics connection-key, the representative indicators defined from the macro perspective are the number of logistics industry employees, the freight turnover and the density of the grade highway network, which were used to investigate the impact of the logistics industry on the comprehensive technical efficiency of manufacturing industry, the change rate of green total factor productivity (MI) in manufacturing industry and even the two-industry-linkage. The logic framework diagram of the full text design from the perspective of integration field as shown in Fig 1.

## Methodology

### Research method

#### Super-SBM model

The evaluation of the development quality of logistics industry and manufacturing industry is mostly based on DEA model, in this paper, input-output efficiency is used as an index to measure the level of high-quality development. SBM (Slack-based Measure) model, that is, the non-radial DEA model based on the measure of relaxation variables, which brings the relaxation variables of input and output into the model function, overcomes the disadvantage that the traditional DEA model does not consider the relaxation of variables. The expression of Super-SBM model considering unexpected output derived from SBM model is:

minρ=1m∑i=1mx¯xik1r1+r2(∑s=1r1yd¯yskd+∑q=1r2yu¯yqku) (1)

$$s.t.\left\{ \begin{array}{c} \bar{x}\geq \sum_{j=1,\neq k}^{n}x_{ij}\lambda_{j}\\ \bar{y^{d}}\leq \sum_{j=1,\neq k}^{n}y_{sj}^{d}\lambda_{j}\\ \bar{y^{u}}\geq \sum_{j=1,\neq k}^{n}\lambda_{j}y_{qj}^{u}\\ \lambda_{j}>0\\ \bar{x}\geq x_{k}\\ \bar{y^{d}}\leq y_{k}^{d}\\ \bar{y^{u}}\leq y_{k}^{u}\\ i=1,\cdots ,m,j=1,\cdots ,n,s=1,\cdots ,r_{1},q=1,\cdots ,r_{2} \end{array}\right.$$

where, *ρ* is the efficiency value, when *ρ*≥1, the decision-making unit is valid; when 0≤*ρ*≤1, the decision-making unit is invalid, indicating that the decision-making unit has efficiency loss. λ is the weight vector, *x*, *y*^*d*^, *y*^*u*^ are the relaxation variables of input, expected output and unexpected output respectively.

#### Malmquist index

Malmquist index (MI) can reflect the change of efficiency over time in different periods, that is, relative to the change of green total factor productivity in the previous year, the MI from *t* to *t* + 1 can be expressed as:

$$M_{0}={\left[\frac{D_{0}^{t}(x_{t+1},y_{t+1})}{D_{0}^{t}(x_{t},y_{t})}\times \frac{D_{0}^{t+1}(x_{t+1},y_{t+1})}{D_{0}^{t+1}(x_{t},y_{t})}\right]}^{\frac{1}{2}}$$ (2)

where, (*x*_*t*_, *y*_*t*_) represents the input-output vector in period *t*, $D_{0}^{t}(x_{t},y_{t})$ represents the distance function. If *M*_0_>1, it indicates that the efficiency of the decision-making unit is improved from *t* to *t* + 1; Otherwise, the efficiency of the decision-making unit decreases; If *M*_0_ = 1, it indicates that the efficiency of the decision-making unit has not changed.

Malmquist index can be divided into two parts: technical progress index (Techch) and technical efficiency index (Effch), as shown in formula (3):

$$M_{0}=Techch\times Effch$$ (3)

where, the technical progress index (Techch) refers to the impact of the progress of production technology on efficiency, and its value is greater than 1, indicating the emergence of technical progress; Otherwise, it means retrogression; If it is 1, it means unchanged. The technical efficiency index (Effch) reflects the improvement of management level, and its value is greater than 1, indicating that the decision-making unit is close to the efficiency frontier. The technical efficiency index (Effch) can be further divided into pure technical efficiency change index (Pech) and scale efficiency change index (Sech):

Effch=Pech×Sech (4)

If Pech is greater than 1, it indicates the progress of pure technical efficiency; On the contrary, it means retrogression; If it is 1, it means unchanged. If Sech is greater than 1, it means that phase *t*+1 is closer and closer to the fixed scale return than phase *t*, or gradually approaches the long-term optimal scale; On the contrary, it means that it is getting farther and farther away.

#### Coupling coordination model

The coupling coordination degree was derived from the capacity coupling coefficient model in physics [62]. It is an index used to evaluate the development level of coupling coordination among systems, reflecting the level of organic combination, interaction between systems. The greater the value, the higher the degree of coupling coordination between systems. In this paper, it will be used to express the two-industry-linkage. The calculation formula is as follows:

$$D=\sqrt{C∙(0.5{∙U}_{1}+0.5∙U_{2})}$$ (5)

where, *U*_1_, *U*_2_ represent the high-quality development index of logistics industry and the high-quality development index of manufacturing industry respectively. *C* represents the coupling degree, which can be used to measure the interaction and influence of multiple systems and reflect the connection strength of each system. Its calculation formula is as follows:

$$C=\sqrt{\frac{U_{1}∙U_{2}}{({\frac{U_{1}+U_{2}}{2})}^{2}}}$$ (6)

(0.5∙*U*_1_+0.5∙*U*_2_) is often called coordination degree T, which refers to the degree of benign coupling in the interaction, reflects the quality of coordination, and can represent whether they promote each other at a high level or restrict each other at a low level. The measurement results are analyzed according to the coupling coordination degree classification standard shown in Table 1.

**Table 1:** Classification standard of coupling coordination degree.

D-value interval of coupling coordination degree	Coordination level	Coupling coordination degree
(0.0~0.1)	1	Extreme disorder
[0.1~0.2)	2	Severe disorder
[0.2~0.3)	3	Moderate disorder
[0.3~0.4)	4	Mild disorder
[0.4~0.5)	5	Verge of disorder
[0.5~0.6)	6	Reluctantly coordination
[0.6~0.7)	7	Primary coordination
[0.7~0.8)	8	Intermediate coordination
[0.8~0.9)	9	Good coordination
[0.9~1.0)	10	High quality coordination

#### PVAR model

Panel Vector Autoregression (PVAR) was proposed on the basis of Vector Autoregressive model (VAR) [63], it can analyze panel data and the advantages of VAR model, all variables are regarded as endogenous variables and analyze the relationship between each variable and its lag term. PVAR model allows data to have individual effects and heteroscedasticity, it cannot only increase the degree of freedom of observations and control individual heterogeneity, but also relax the time stability requirements of data, so as to explain the complex relationship between variables [64]. In view of this, this paper uses PVAR model to analyze the interaction between logistics industry and manufacturing industry [30]. The PVAR model is as follows:

$$y_{it}=\sum_{j=1}^{m}\beta_{j}y_{it-j}+\mu_{i}{+\beta }_{e,t}+\varepsilon_{it}$$ (7)

where, *y*_*it*_ represents the column vector of the investigated variable, *i* and *t* represent province and time respectively; *m* represents lag order, *β*_*j*_ represents the coefficient of the corresponding lag term, and the value represents its effect on *y*_*it*_, *μ*_*i*_ represents the individual fixed effects vector, *β*_*e*,*t*_ represents the time effects vector, *ε*_*it*_ is a random perturbation term.

According to the theory of integration field, the basic structure of logistics network chain is composed of logistics base-nuclear, logistics integrator and logistics connection-key. The logistics base-nuclear is the carrier of the field source, which mainly plays the role of attracting the integrator, and the freight turnover represents the operation capacity of the base-nuclear, so the freight turnover was used to represent its capacity; Logistics integrator is usually the leading logistics enterprise in the supply chain and logistics chain, which dominates the formation of supply chain or logistics chain, therefore, the number of logistics industry employees was used to represent the scale of logistics integrator. There are many types of logistics connection-keys, and a very important role of it is to connect various business entities in the logistics chain and supply chain, therefore, the level of logistics connection-keys can be represented by the density of grade highway network. Relevant indicators of PVAR model are shown in Table 2.

**Table 2:** PVAR model index description.

Indexes	Variable symbol	Index measurement	Unit
Comprehensive technical efficiency of manufacturing industry	gyzhxl	Comprehensive technical efficiency of manufacturing industry	-
Change rate of green total factor productivity in manufacturing industry	gymi	Manufacturing industrial MI	-
Comprehensive technical efficiency of logistics industry	wlzhxl	Comprehensive technical efficiency of logistics industry	-
Change rate of logistics green total factor productivity	wlmi	Logistics industry MI	-
Efficiency of the two-industry-linkage	wgd	Coupling coordination degree of manufacturing industry and logistics industry	-
Capability of logistics base-nuclear	zzl	Freight turnover	100 million ton km
Scale of logistics integrator	wlry	Number of logistics industry employees	ten thousand people
Level of logistics connection-key	djglmd	Density of grade highway network	km / km^2^

### Data source and description

Because the data of Hong Kong, Macao, Taiwan and Tibet are partially missing, this paper selected the data of the other 30 provinces, autonomous regions, municipalities and cities in China as the research sample, with a time span of 2001–2019. The data are mainly from China Statistical Yearbook, China Energy Statistical Yearbook and China Industrial Statistical Yearbook. The original data of carbon emission mainly comes from the carbon emission inventories from 2001 to 2018 provided by CEADs database, which is the most authoritative statistical data for accounting China’s carbon emission level at present. In particular, due to the lack of manufacturing data, but the actual manufacturing accounts for more than 70% of the industry, replace manufacturing data with industrial data. If some original data are missing, they shall be supplemented by interpolation method, and finally 13 indicators were selected. The description and statistics of core variables are shown in Table 3.

**Table 3:** Descriptive statistics of core variables.

Dimension	Variables	Sample Size	Min	Max	Mean	Standard Deviation
Logistics industry investment	Fixed asset investment in logistics industry	570	29.990	3364.121	644.115	608.479
	Number of logistics industry employees	570	2.800	86.400	23.546	14.244
	Energy consumption of logistics industry	570	23.913	3559.573	848.715	641.550
Logistics output	Added value of logistics industry	570	19.500	3776.720	705.750	649.720
	Carbon emission of logistics industry	570	0.500	70.545	16.452	12.699
Manufacturing investment	Net value of fixed assets of manufacturing industry	570	182.010	26933.180	5130.682	4583.304
	Number of manufacturing industry employees	570	9.700	1568.000	277.569	302.670
	Energy consumption of manufacturing industry	570	168.166	23332.300	5867.241	4474.654
Manufacturing output	Added value of manufacturing industry	570	58.000	36973.730	4621.348	5682.248
	Carbon emissions of manufacturing industry	570	5.300	1038.870	217.133	172.603
Capability of logistics base-nuclear	Freight turnover	570	97.5	30324.9	4043.584	4645.409
Scale of logistics integrator	Number of logistics industry employees	570	2.8	86.4	23.546	14.244
Level of logistics connection-key	Density of grade highway network	570	0.024	2.098	0.667	0.476

## Results

Firstly, MAXDEA ultra 7.10 software was used to obtain the comprehensive technical efficiency of logistics industry and manufacturing industry, and the change rate of green total factor productivity (MI) of them, then SPSSAU was used to analyze the coupling coordination degree of the two industries, and then stata16.0 was used to analyze the interaction between the three elements of logistics industry and the comprehensive technical efficiency of manufacturing industry (gyzhxl), and the interaction with the green total factor productivity change rate of manufacturing industry (gymi), finally, the impact of the three elements of logistics on the two-industry-linkage was analyzed.

### Interaction between logistics industry and the comprehensive technical efficiency of manufacturing industry from the perspective of integration field

#### Unit root test of variables

In order to avoid the violent fluctuation of data and eliminate the possible heteroscedasticity, the relevant time series data were logarithmicized. The processed data are the logarithm of the comprehensive technical efficiency of manufacturing industry(lngyzhxl), the logarithm of freight turnover (lnzzl), the logarithm of the number of logistics industry employees (lnwlry) and the logarithm of the density of grade highway network (lndjglmd). The unit root stationarity test was carried out for the variable sequence, and HT and IPS were selected as the test standards [65], the test results using stata16.0 software are shown in Table 4.

**Table 4:** Description of unit root test results of variables.

Variables	Inspection method	Statistic	p-value	Conclusion
lngyzhxl	HT	0.5831	0.0685	stable
	IPS	-3.6682	0.0001	stable
lnzzl	HT	0.5169	0.0007	stable
	IPS	-1.3768	0.0843	stable
lnwlry	HT	0.5913	0.0998	stable
	IPS	-4.4015	0.0000	stable
lndjglmd	HT	0.7002	0.9231	unstable
	IPS	-1.8715	0.0306	stable

The test results show that some sequences are non-stationary at the significance level of 10%. Then, the first-order difference sequence was tested and the results shown in Table 5 were obtained.

**Table 5:** Unit root test results of variables first-order difference sequences.

Variables	Inspection method	Statistic	p-value	Conclusion
d_lngyzhxl	HT	-0.1288	0.0000	stable
	IPS	-11.6494	0.0000	stable
d_lnzzl	HT	-0.1739	0.0000	stable
	IPS	-9.3001	0.0000	stable
d_lnwlry	HT	-0.0154	0.0000	stable
	IPS	-11.6296	0.0000	stable
d_lndjglmd	HT	0.1008	0.0000	stable
	IPS	-9.7387	0.0000	stable

The test results in Table 5 show that under the significance level of 1%, the first-order difference sequences are stable, i.e. I(1). Cointegration theory [66] shows that although variables have their own long-term fluctuation laws, if they are cointegrated of order (d, d), there is a long-term stable proportional relationship between them. Some variables in the original data series are unstable, according to the above variable unit root test, all variables (lngyzlxl, lnzzl, lnwlry, lndjglmd) belong to first-order cointegration. According to cointegration theory, the comprehensive technical efficiency of manufacturing industry (lngyzhxl) and logistics variables (lnzzl, lnwlry, lndjglmd) constitute a long-term equilibrium relationship, Kao test, Pedroni test and Westerlund test were used to test the cointegration relationship between variables to investigate whether there is a long-term equilibrium cointegration relationship between variables. The test results are shown in Table 6, most of the tests reject the original test without cointegration relationship, that is, it can be judged that there is cointegration relationship between variables.

**Table 6:** Cointegration test of logistics industry and the comprehensive technical efficiency of manufacturing industry.

Inspection method	Index	Statistic	p-value
Kao test	Modified Dickey-Fuller t	-1.4045	0.0801
	Dickey-Fuller t	-2.5462	0.0054
	Augmented Dickey-Fuller t	-0.2759	0.3913
	Unadjusted modified Dickey Fuller t	-1.3389	0.0903
	Unadjusted Dickey-Fuller t	-2.5095	0.0060
Pedroni test	Modified Phillips-Perron t	2.0920	0.0182
	Phillips-Perron t	-6.0233	0.0000
	Augmented Dickey-Fuller t	-5.2765	0.0000
Westerlund test	Variance ratio	-1.9856	0.0235

#### Determine the optimal lag order

The PVAR model for logistics industry and the comprehensive technical efficiency of manufacturing industry was established, first determine the lag order, select it by using Lian Yujun’s stata16.0 software package pvar2, and determine the lag period according to the minimum criteria such as AIC and SC, the results are shown in Table 7.

**Table 7:** Lag test of PVAR model for logistics industry and the comprehensive technical efficiency of manufacturing industry.

lag	AIC	BIC	HQIC
1	-5.17878	-3.99621	-4.71394
2	-5.39372	-4.00571	-4.84665
3	-5.32685	-3.71075	-4.68809
4	-5.17897	-3.30776	-4.43721
5	-6.89553*	-4.73658*	-6.03709*

It can be seen from Table 7 that the lag period of the PVAR model for the interaction between the comprehensive technical efficiency of logistics industry and manufacturing industry is 5, the PVAR(5) model was established by stata16.0 software, and the estimation results are shown in Table 8. However, some scholars believed that PVAR model is a pan theoretical model, and the positive and negative, magnitude and significance of its parameter estimates lack practical economic significance [67], it cannot describe the long-term impact mechanism, evolution path and impact degree of one variable on other variables [68], therefore, this paper only gives the estimation results (Table 8), and the analysis focuses on the subsequent impulse response and variance decomposition.

**Table 8:** GMM estimation results of PVAR model for logistics industry and the comprehensive technical efficiency of manufacturing industry.

	h_d_lngyzhxl	h_d_lnzzl	h_d_lnwlry	h_d_lndjglmd
L.h_d_lngyzhxl	-0.1319	0.0205	0.0223	0.0597**
L.h_d_lnzzl	-0.0164	-0.1415	0.0540**	0.005
L.h_d_lnwlry	-0.0281	-0.0733	-0.0057	-0.0126
L.h_d_lndjglmd	-0.0215	0.1950**	0.0155	0.1493***
L2.h_d_lngyzhxl	0.1071**	0.0514	-0.047	0.0647**
L2.h_d_lnzzl	-0.029	0.0602	0.0376	0.0233**
L2.h_d_lnwlry	0.0159	-0.3171**	0.0275	0.0016
L2.h_d_lndjglmd	-0.0394	0.6117***	-0.0687**	0.0640**
L3.h_d_lngyzhxl	0.063	0.2780**	-0.2345***	0.0832***
L3.h_d_lnzzl	-0.0172	0.0051	0.0822***	0.0210*
L3.h_d_lnwlry	0.0433	-0.0443	0.0433	-0.0047
L3.h_d_lndjglmd	0.0288	-0.0096	-0.0640*	0.0295
L4.h_d_lngyzhxl	0.0861**	0.1076	0.0094	0.0876***
L4.h_d_lnzzl	0.008	0.0129	0.0396	0.0314***
L4.h_d_lnwlry	-0.0707*	0.0764	0.0698*	-0.0734***
L4.h_d_lndjglmd	-0.0953**	0.1284*	-0.0178	-0.0391*
L5.h_d_lngyzhxl	-0.0394	-0.0938	0.0389	-0.0155
L5.h_d_lnzzl	-0.0121	0.2305***	0.1346***	0.0200*
L5.h_d_lnwlry	-0.0384	0.014	0.0034	-0.023
L5.h_d_lndjglmd	-0.0085	0.1585**	-0.0096	-0.0013

Note

* ** *** represents the significance levels of 10%,5% and 1% respectively.

where, h_ represents the variable after eliminating the fixed effect by Helmert transform; L1, L2, L3, L4, L5 indicate lag first order to lag fifth order.

In the results shown in Table 8, when the explanatory variable is the comprehensive technical efficiency of the manufacturing industry, its own lag first-order and fifth-order has insignificant negative effects, lag second-order and fourth-order have significant positive effects, and lag third-order has insignificant positive effects, which is manifested as inhibition–significant promotion–promotion–significant promotion–inhibition; The first, second, third and fifth order lag of freight turnover has insignificant negative effect, and the fourth-order lag has insignificant positive effect, which is manifested as inhibition–inhibition–inhibition–promotion–inhibition; The first-order and fifth-order lag of the number of logistics industry employees has insignificant negative effect, the second-order and third-order lag have insignificant positive effect, and the fourth-order lag has significant negative effect, which is manifested as inhibition–promotion–promotion–significant inhibition–inhibition; The first, second and fifth order lag of the density of the grade highway network has insignificant negative effect, the third-order lag has insignificant positive effect, and the fourth-order lag has significant negative effect, which is manifested as inhibition–inhibition–promotion–significant inhibition–inhibition. When the explained variable is freight turnover, its first-order to fifth-order lag is inhibition–promotion–promotion–promotion–significant promotion; The comprehensive technical efficiency of manufacturing industry is represented by promotion–promotion–significant promotion–promotion–inhibition; The number of logistics industry employees shows inhibition–significant inhibition–inhibition–promotion–promotion; The density of the grade highway network shows significant promotion–significant promotion–inhibition–significant promotion–significant promotion.

When the explained variable is the number of logistics industry employees, the first-order to fifth-order lag is inhibition–promotion–promotion–significant promotion–promotion; The comprehensive technical efficiency of manufacturing industry shows promotion–inhibition–significant inhibition–promotion–promotion; The freight turnover shows significant promotion–promotion–significant promotion–promotion–significant promotion; The density of the grade highway network shows promotion–significant inhibition–significant inhibition–inhibition–inhibition. When the explained variable is the density of the grade highway network, its own lag from first-order to fifth-order is significant promotion–significant promotion–significant inhibition–inhibition–inhibition; The comprehensive technical efficiency of manufacturing industry shows significant promotion–significant promotion–significant promotion–significant promotion–inhibition; The freight turnover shows promotion–significant promotion–significant promotion–significant promotion–significant promotion; The number of logistics industry employees shows inhibition–promotion–inhibition–significant inhibition–inhibition.

#### Stability of the model and Granger causality test

The optimal lag order was set as 5 to further verify the model’s stability of logistics industry and the comprehensive technical efficiency of manufacturing industry and the Granger causality between variables, all characteristic roots (including real roots and virtual roots) are less than 1. Therefore, the PVAR model is stable and passes the stability test. Then, Granger causality test was carried out for each variable with a lag of 5 periods [69], and the results shown in Table 9 were obtained.

**Table 9:** Granger causality test of logistics industry and the comprehensive technical efficiency of manufacturing industry.

Original hypothesis: the latter is not the Granger reason of the former			p-value	Conclusion
h_d_lngyzhxl	-	h_d_lnzzl	0.920	supported
h_d_lngyzhxl	-	h_d_lnwlry	0.383	supported
h_d_lngyzhxl	-	h_d_lndjglmd	0.206	supported
h_d_lnzzl	-	h_d_lngyzhxl	0.164	supported
h_d_lnzzl	-	h_d_lnwlry	0.056	rejected
h_d_lnzzl	-	h_d_lndjglmd	0.000	rejected
h_d_lnwlry	-	h_d_lngyzhxl	0.007	rejected
h_d_lnwlry	-	h_d_lnzzl	0.000	rejected
h_d_lnwlry	-	h_d_lndjglmd	0.067	rejected
h_d_lndjglmd	-	h_d_lngyzhxl	0.003	rejected
h_d_lndjglmd	-	h_d_lnzzl	0.012	rejected
h_d_lndjglmd	-	h_d_lnwlry	0.034	rejected

It can be seen from Table 9 that the freight turnover, the number of logistics industry employees and the density of the grade highway network are not Granger reasons for the comprehensive technical efficiency of manufacturing industry, and the comprehensive technical efficiency of manufacturing industry is not Granger reasons for the freight turnover. The number of logistics industry employees and the density of the grade highway network are Granger reasons for the freight turnover. The comprehensive technical efficiency of manufacturing industry, the freight turnover, and the density of the grade highway network are Granger reasons of the number of logistics industry employees. The comprehensive technical efficiency of manufacturing industry, freight turnover and the number of logistics industry employees are the Granger reasons of the density of the grade highway network. It shows that the current scale of major logistics integrator, the level of logistics connection-key and the capacity of logistics base-nuclear have no obvious impact on the comprehensive technical efficiency of manufacturing industry, or their early changes cannot effectively explain the changes in the comprehensive technical efficiency of manufacturing industry (the main reason is that Granger causality test focuses on whether one variable has the ability to predict another variable, the availability of relevant data may weaken the test results, and the development of logistics is relatively slow, resulting in the absence of Granger causality), and the comprehensive technical efficiency of manufacturing industry mainly affects the scale of logistics integrator and the level of logistics connection-key. Within the logistics industry, the interaction effect of the capacity of logistics base-nuclear, the scale of logistics integrator and the level of logistics connection-key are obvious.

#### PVAR impulse response analysis

**Fig 2:**
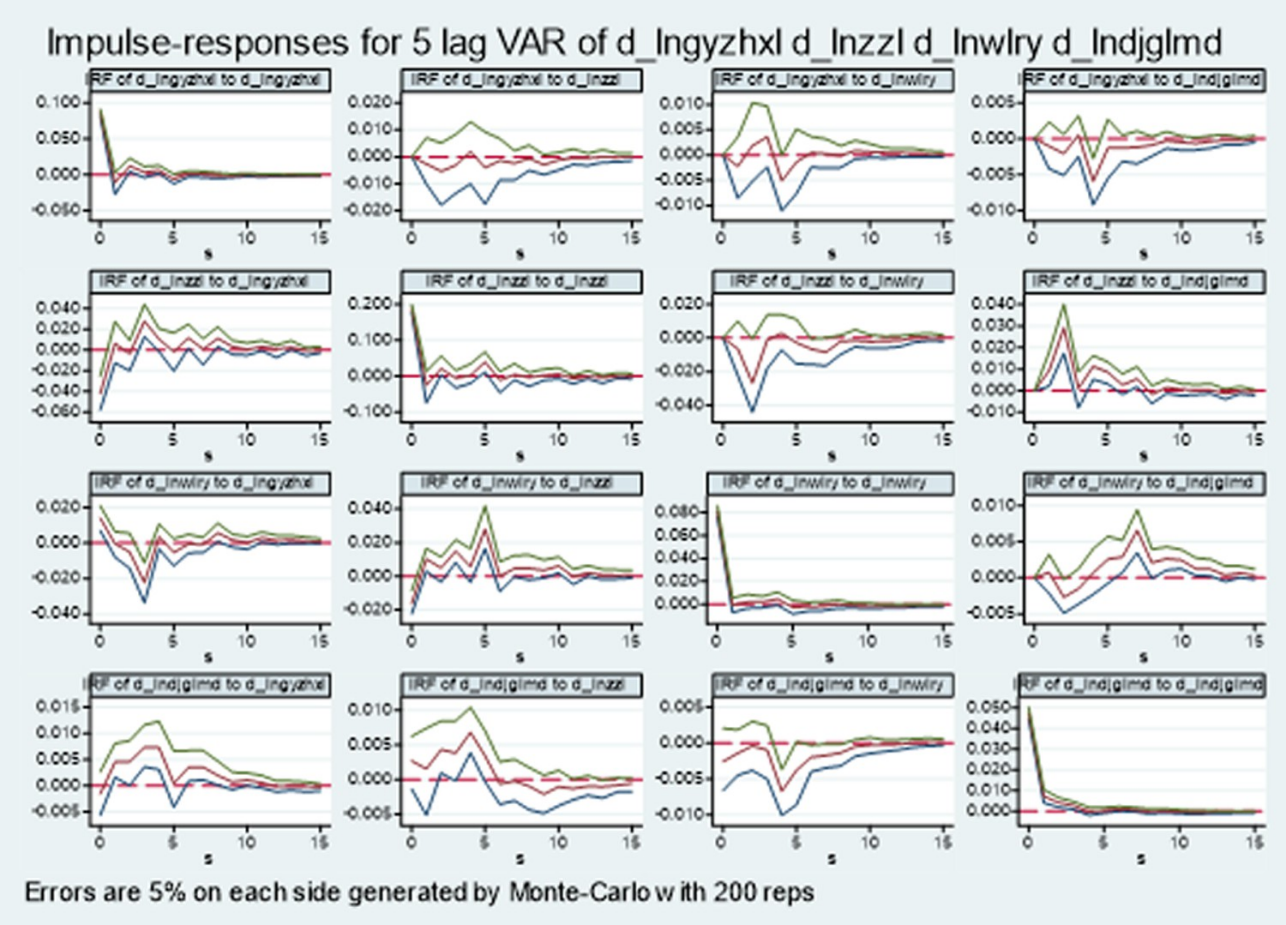
Impulse response diagram of coupling and coordinated development of two industries.

In order to fully describe the long-term dynamic impact effect between the logistics industry and the comprehensive technical efficiency of manufacturing industry, the impulse response function was further analyzed, and the 95% confidence interval impulse response diagram of other endogenous variables was calculated 200 times by Monte Carlo random simulation, as shown in Fig 2. The horizontal axis represents the number of response periods, the vertical axis represents the magnitude of impulse response value, the upper and lower lines represent the confidence interval of 5% ~ 95%, and the middle line represents the estimation curve of impulse response function.

For a standard deviation impact from the comprehensive technical efficiency of manufacturing industry itself, it immediately produces a strong positive response, reaches the peak, then decreases rapidly, becomes negative in the first lag period and begins to converge to 0, indicating that the comprehensive technical efficiency of manufacturing industry is highly dependent on itself, but this inertia gradually weakens. For a standard deviation impact from the freight turnover, the comprehensive technical efficiency of manufacturing industry shows a negative response at the beginning, becomes positive in the first lag period, and then reaches the peak in the fourth lag period, after that, the lag begins to weaken and converge to 0. This also shows that in the process of increasing the freight turnover, the increase of some invalid transportation distance and transportation times is not conducive to the high-quality development of industry and even economy, considering the capacity of logistics base-nuclear, blindly exceeding the demand of the capacity of logistics base-nuclear will cause a waste of resources, but not conducive to the high-quality development of industry, it is also not conducive to high-quality economic development. The matching between the development of the capability of logistics base-nuclear and the high-quality development of industry needs to go through a certain stage of practice and running in, when there is an error matching between the two industries, the capability of logistics base-nuclear will inhibit the high-quality development of industry. For a standard deviation impact from the number of logistics industry employees, the comprehensive technical efficiency of manufacturing industry has a positive response at the beginning, and then gradually decreases, it reaches a negative bottom in the third lag period, and is positive in the fourth lag period, and then converges to 0 in the fluctuation. This shows that the increase of the number of logistics industry employees has a positive impact on the comprehensive technical efficiency of manufacturing industry in the short term, but excessive increase will cause a waste of human resources, the emergence or expansion of the scale of logistics integrator is more to meet the needs of the market, it takes a period of market running in to show a positive promotion of high-quality industrial development. The impact of a standard deviation impact from the density of the grade highway network on the comprehensive technical efficiency of manufacturing industry is reverse at first, then increases, reaches the peak in the fourth lag period, and then gradually weakens and converges to 0. In the early stage, the increase of the density of the grade highway network and even the improvement of the level of logistics connection-key have driven the respective development of the two industries, and even the high-quality development of the industry, with the gradual improvement of the high-quality development of the two industries in the later stage, the optimization of the linkage system of the two industries has been gradually realized.

For the freight turnover, a standard deviation impact from the comprehensive technical efficiency of manufacturing industry fluctuates slightly below 0 and converges to 0 in the lag period 1 ~ 5, which also confirms that the improvement of the comprehensive technical efficiency of manufacturing industry may require more efficient logistics turnover. The impact of freight turnover on its own standard deviation reached a positive peak at the beginning, and then weakened rapidly and converged to 0. This also shows that the rapid expansion of the capacity of logistics base-nuclear depends on itself, but the long-term development also depends on the influence of the outside world. A standard deviation impact of the number of logistics industry employees on the freight turnover was negative at first, then increased to positive in the second lag period, reached the peak in the fifth lag period, and the lag gradually converged to 0, the overall performance is positive, which also shows the important role of the number of logistics industry employees in completing the freight turnover, at the same time, it further explains the further demand impact of the expansion of the scale of logistics integrator on the logistics base-nuclear capability. A standard deviation impact of the density of the highway network on the freight turnover is positive at the beginning, and maintains a relatively high positive value, it reaches the peak in the fourth lag period, and then gradually becomes negative and converges to 0, indicating that when the density of the highway network increases, the freight turnover is more convenient. The impact of logistics connection-key on the capability of logistics base-nuclear is also positive in an appropriate range, but the continuous development of logistics connection-key is not necessarily conducive to the expansion of the capability of logistics base-nuclear, such as the increase of the density of the grade highway network may lead to more fierce competition between logistics base-nuclear.

For the number of logistics industry employees, the impact of a standard deviation impact from the comprehensive technical efficiency of manufacturing industry mainly fluctuates up and down at 0, reaching the peak in the third lag period, reaching the trough in the fourth lag period, and then rising and converging to 0. The increase of the number of logistics industry employees is reflected in the increase of investment in the high-quality development of the logistics industry, thus reducing the efficiency of high-quality logistics, the impact of the increase of the scale of logistics integrator on the comprehensive technical efficiency of manufacturing industry is positive in a certain range, but beyond the normal range, it may have negative effects. A standard deviation impact of freight turnover on the number of logistics industry employees was negative at first, reached the trough in the second period, and then gradually converged to 0. The increase of freight turnover may lead to the demand for mechanization, thus reducing the demand for the number of logistics industry employees, and the increase of the capacity of logistics base-nuclear requires a corresponding logistics integrator. The impact of a standard deviation of the number of logistics industry employees on themselves reached the peak at the beginning, and then rapidly decreased and converged to 0, which also shows that the number of logistics industry employees are more dependent on themselves, and the development of logistics integrator is more dependent on their own ability and even resources. A standard deviation impact of the density of the grade highway network on the number of logistics industry employees was negative at first, then basically fluctuated below 0, reached the trough after lagging behind phase 5, and gradually converged to 0. There is no doubt that the increase of the density of the grade highway network is conducive to improving the efficiency of logistics industry, but efficient logistics industry means the improvement of mechanization level and the reduction of labor demand rate. The development of logistics connection-key will also be conducive to the development of logistics integrator, such as expanding business scope and improving service level.

For the density of the grade highway network, the impact of a standard deviation impact from the comprehensive technical efficiency of manufacturing industry mainly fluctuates below 0, which shows that the positive impact of the comprehensive technical efficiency of manufacturing industry on the density of the grade highway network is not obvious. A standard deviation impact of freight turnover on the density of the grade highway network is positive, and reaches the peak in the second lag period, and then converges to 0 in the fluctuation, the increase of freight turnover is a main reason for the increase of the density of the grade highway network. This also shows that the increase of the capacity of logistics base-nuclear is a main reason for the further development of logistics connection-key. A standard deviation impact of the number of logistics industry employees on the density of the grade highway network is not obvious at the beginning, it reaches the trough in the second lag period, then gradually rises, reaches the peak in the seventh lag period, and then slowly decreases and converges to 0. The increase of the number of logistics industry employees means the increase of the scale of logistics integrator, thus, the increase of the density of the grade highway network is accompanied by the improvement of the level of logistics connection-key. The impact of the density of the grade highway network on its own standard deviation is positive at the beginning, and then rapidly converges to 0 in the first lag period, which also shows that the density of the grade highway network needs to rely more on the development of external factors.

#### Variance decomposition

The variance decomposition of PVAR is a further supplement to impulse response analysis, it mainly describes the firmness of each structural impact on the variables in the system, and can specifically analyze the contribution of each variable to more accurately measure the degree of interaction between variables. The variance decomposition results of the interaction between the three elements of logistics industry and the comprehensive technical efficiency of manufacturing industry are shown in Table 10.

**Table 10:** Variance decomposition of logistics industry and the comprehensive technical efficiency of manufacturing industry.

Variables	Phase	d_lngyzhxl	d_lnzzl	d_lnwlry	d_lndjglmd
d_lngyzhxl	1	1.000	0.000	0.000	0.000
d_lngyzhxl	2	0.998	0.001	0.001	0.000
d_lngyzhxl	3	0.994	0.005	0.001	0.001
d_lngyzhxl	4	0.991	0.006	0.003	0.001
d_lngyzhxl	5	0.983	0.006	0.006	0.005
d_lngyzhxl	10	0.978	0.010	0.006	0.006
d_lngyzhxl	15	0.977	0.011	0.006	0.006
d_lngyzhxl	20	0.977	0.011	0.006	0.006
d_lngyzhxl	25	0.977	0.011	0.006	0.006
d_lngyzhxl	30	0.977	0.011	0.006	0.006
d_lnzzl	1	0.048	0.952	0.000	0.000
d_lnzzl	2	0.048	0.949	0.001	0.002
d_lnzzl	3	0.046	0.912	0.019	0.024
d_lnzzl	4	0.064	0.894	0.019	0.023
d_lnzzl	5	0.066	0.889	0.019	0.026
d_lnzzl	10	0.069	0.882	0.021	0.027
d_lnzzl	15	0.070	0.882	0.021	0.027
d_lnzzl	20	0.070	0.882	0.021	0.027
d_lnzzl	25	0.070	0.882	0.021	0.027
d_lnzzl	30	0.070	0.882	0.021	0.027
d_lnwlry	1	0.028	0.038	0.934	0.000
d_lnwlry	2	0.028	0.052	0.920	0.000
d_lnwlry	3	0.031	0.055	0.913	0.001
d_lnwlry	4	0.091	0.078	0.830	0.001
d_lnwlry	5	0.092	0.081	0.826	0.001
d_lnwlry	10	0.089	0.166	0.737	0.009
d_lnwlry	15	0.090	0.169	0.731	0.010
d_lnwlry	20	0.090	0.169	0.731	0.010
d_lnwlry	25	0.090	0.169	0.731	0.010
d_lnwlry	30	0.090	0.169	0.731	0.010
d_lndjglmd	1	0.001	0.003	0.003	0.993
d_lndjglmd	2	0.010	0.004	0.004	0.983
d_lndjglmd	3	0.018	0.012	0.004	0.967
d_lndjglmd	4	0.039	0.017	0.004	0.940
d_lndjglmd	5	0.057	0.034	0.021	0.888
d_lndjglmd	10	0.067	0.039	0.029	0.865
d_lndjglmd	15	0.067	0.041	0.029	0.863
d_lndjglmd	20	0.067	0.042	0.029	0.862
d_lndjglmd	25	0.067	0.042	0.029	0.862
d_lndjglmd	30	0.067	0.042	0.029	0.862

The results in Table 10 show that the variance contribution rate of the comprehensive technical efficiency of manufacturing industry to itself tends to be stable from 100% lagging behind in phase 1 to 97.7% in phase 15, indicating that the comprehensive technical efficiency of manufacturing industry is its biggest contribution factor. The contribution of freight turnover, the number of logistics industry employees and the density of the grade highway network to the comprehensive technical efficiency of manufacturing industry was relatively small at the beginning and then increased slightly, the freight turnover increased to 1.1% in the 15th lag period, the number of logistics industry employees increased to 0.6% in the 5th lag period, and the density of the grade highway network increased to 0.6% in the 10th lag period. It can be seen from the perspective of logistics industry, from large to small, the contribution to the comprehensive technical efficiency of manufacturing industry is the capacity of logistics base-nuclear, the scale of logistics integrator and the level of logistics connection-key, but the role is relatively small.

The variance contribution rate of freight turnover to itself tends to be stable from 95.2% in phase 1 to 88.2% in phase 10, indicating that freight turnover is its biggest contribution factor. However, the contribution of the comprehensive technical efficiency of manufacturing industry, the number of logistics industry employees and the density of the grade highway network to the development of freight turnover is relatively small, but their contributions are gradually increasing. The contribution of the comprehensive technical efficiency of manufacturing industry reached 7% in the 15th lag period, and the contribution of the number of logistics industry employees and the density of the grade highway network increased to 2.1% and 2.7% respectively in the 10th lag period. It can be seen that the contribution to the development of the capability of logistics base-nuclear is the comprehensive technical efficiency of manufacturing industry, the level of logistics connection-key and the scale of logistics integrator.

The variance contribution rate of the number of logistics industry employees to themselves began to stabilize from 92% in the first lag period to 73.1% in the 15th lag period, indicating that the number of logistics industry employees are their biggest contribution factor. The comprehensive technical efficiency of manufacturing industry, freight turnover and the density of the grade highway network have made relatively small contributions to the number of logistics industry employees, but their contributions are gradually increasing. The manufacturing industry’s comprehensive technical efficiency, freight turnover and the density of the grade highway network have increased to 9%, 16.1% and 1% respectively in the 15th period. From large to small, the contributions to the scale of logistics integrator are the capacity of logistics base-nuclear, the comprehensive technical efficiency of manufacturing industry and the level of logistics connection-key.

The contribution rate of the density of the grade highway network to its own variance tends to be stable from 99.3% in the first stage to 86.2% in the 20th stage, indicating that the density of the grade highway network is its biggest contribution factor. However, the contribution of the comprehensive technical efficiency of manufacturing industry, freight turnover and the number of logistics industry employees to the density of the grade highway network is relatively small, but their contributions are gradually increasing. The comprehensive technical efficiency of manufacturing industry and the number of logistics industry employees increased to 6.7% and 2.9% respectively in the 10th lag period, and the freight turnover increased to 4.2% in the 20th lag period. From the perspective of logistics industry, the contribution to the development of the level of logistics connection-key from large to small is the comprehensive technical efficiency of manufacturing industry, the capacity of logistics base-nuclear and the scale of logistics integrator.

In the long run, the impact of the comprehensive technical efficiency of manufacturing industry on other factors from large to small is the number of logistics industry employees, freight turnover, the density of the grade highway network, that is, the scale of logistics integrator, the capacity of logistics base-nuclear and the level of logistics connection-key. The influence of freight turnover on other factors from large to small is the number of logistics industry employees, the density of the grade highway network and the comprehensive technical efficiency of manufacturing industry, that is, the influence of the capacity of logistics base-nuclear on other factors from large to small is the scale of logistics integrator, the level of logistics connection-key and high-quality development level of manufacturing industry. The influence of the number of logistics industry employees on other factors from large to small is the density of the grade highway network, the freight turnover and the comprehensive technical efficiency of manufacturing industry, that is, the influence of the scale of logistics integrator on other factors from large to small is the level of logistics connection-key, the capacity of logistics base-nuclear and the high-quality development level of manufacturing industry. The influence of the density of the grade highway network on other factors from large to small is freight turnover, the number of logistics industry employees and the comprehensive technical efficiency of manufacturing industry, that is, the influence of the level of logistics connection-key on other factors from large to small is the capacity of logistics base-nuclear, the scale of logistics integrator and high-quality development level of manufacturing industry.

### Interaction between logistics industry and the change rate of green total factor productivity in manufacturing industry from the perspective of integration field

#### Unit root test of variables

In order to avoid the violent fluctuation of data and eliminate the possible heteroscedasticity, the relevant time series data were logarithmicized. The processed data are the logarithm of the change rate of manufacturing green total factor productivity (manufacturing MI) (lngymi), the logarithm of freight turnover (lnzzl), the logarithm of the number of logistics industry employees (lnwlry) and the logarithm of the density of the grade highway network (lndjglmd). The unit root stationarity test was conducted for the variable series, according to the statistical characteristics of the data, HT and IPS were selected as the test standards [65]. The test results by using stata16 software are shown in Table 11.

**Table 11:** Description of unit root test results of variables.

Variables	Inspection method	Statistic	p-value	Conclusion
lngymi	HT	0.1432	0.0000	stable
	IPS	-11.0211	0.0000	stable
lnzzl	HT	0.5169	0.0007	stable
	IPS	-1.3768	0.0843	stable
lnwlry	HT	0.5913	0.0998	stable
	IPS	-4.4015	0.0000	stable
lndjglmd	HT	0.7002	0.9231	unstable
	IPS	-1.8715	0.0306	stable

The test results show that some sequences are non-stationary at the significance level of 10%. Then, the first-order difference sequence was tested and the results shown in Table 12 were obtained.

**Table 12:** Description of unit root test results of variable first-order difference sequence.

Variables	Inspection method	Statistic	p-value	Conclusion
d_lngymi	HT	-0.4982	0.0000	stable
	IPS	-13.9557	0.0000	stable
d_lnzzl	HT	-0.1739	0.0000	stable
	IPS	-9.3001	0.0000	stable
d_lnwlry	HT	-0.0154	0.0000	stable
	IPS	-11.6296	0.0000	stable
d_lndjglmd	HT	0.1008	0.0000	stable
	IPS	-9.7387	0.0000	stable

The test results show that at the significance level of 1%, the first-order difference sequences are stable, i.e. I(1). Similarly, according to the cointegration theory, manufacturing MI (lngymi) and logistics development variables (lnzzl, lnwlry, lndjglmd) form a long-term equilibrium relationship. The results shown in Table 13 were obtained through the cointegration test. The tests reject the original test without cointegration relationship, that is, it can be judged that there is a cointegration relationship between variables.

**Table 13:** Cointegration test of logistics industry and manufacturing MI.

		Statistic	p-value
Kao test	Modified Dickey-Fuller t	-15.1234	0.0000
	Dickey-Fuller t	-39.0954	0.0000
	Augmented Dickey-Fuller t	-16.0384	0.0000
	Unadjusted modified Dickey Fuller t	-34.9794	0.0000
	Unadjusted Dickey-Fuller t	-44.6636	0.0000
Pedroni test	Modified Phillips-Perron t	-2.5935	0.0047
	Phillips-Perron t	-31.9687	0.0000
	Augmented Dickey-Fuller t	-35.7775	0.0000
Westerlund test	Variance ratio	-4.0730	0.0000

#### Determine the optimal lag order

The PVAR model for logistics industry and manufacturing MI was established, first determine the lag order, select it by using Lian Yujun’s stata16.0 software package pvar2, and determine the lag period according to the minimum criteria such as AIC and SC, the results are shown in Table 14.

**Table 14:** Lag test of PVAR model for logistics industry and manufacturing MI.

lag	AIC	BIC	HQIC
1	-4.18751	-2.9456	-3.69803
2	-4.48405	-3.02186	-3.90613
3	-4.83609	-3.1276	-4.15883
4	-6.1778	-4.19157	-5.38804
5	-6.84241*	-4.53993*	-5.92398*

It can be seen from Table 14 that the lag period of PVAR model for logistics industry and manufacturing MI is 5. PVAR(5) model for the two-industry-linkage was established by using Stata software, and the estimation results are shown in Table 15.

**Table 15:** GMM estimation results of PVAR model for logistics industry and manufacturing MI.

	h_d_lngyzhxl	h_d_lnzzl	h_d_lnwlry	h_d_lndjglmd
L.h_d_lngymi	-0.5666***	-0.0040	0.0261	-0.0155
L.h_d_lnzzl	-0.0425	-0.1765	0.0652***	0.0007
L.h_d_lnwlry	0.0247	-0.1283	0.0563	-0.0179
L.h_d_lndjglmd	0.1540	0.8624***	-0.2021**	0.4664***
L2.h_d_lngymi	-0.3314***	-0.2545*	0.1420**	-0.0031
L2.h_d_lnzzl	-0.0664**	0.0514	0.0308	0.0257***
L2.h_d_lnwlry	0.0636	-0.3327**	0.0024	-0.0078
L2.h_d_lndjglmd	-0.0637	0.4545***	-0.0114	0.0192
L3.h_d_lngymi	-0.3582***	-0.0124	0.0447	0.0476*
L3.h_d_lnzzl	-0.0358	-0.0264	0.0799***	0.0157
L3.h_d_lnwlry	0.0947*	-0.0314	0.0560	0.0025
L3.h_d_lndjglmd	0.0261	-0.0310	-0.0806**	-0.0111
L4.h_d_lngymi	-0.2437***	0.0683	0.0452	0.0776
L4.h_d_lnzzl	-0.0686**	-0.0181	0.0327	0.0165*
L4.h_d_lnwlry	-0.0169	0.1181	0.0237	-0.0528**
L4.h_d_lndjglmd	0.1215*	0.0943	0.0312	-0.0570**
L5.h_d_lngymi	-0.1854***	0.0045	-0.0141	0.0396**
L5.h_d_lnzzl	-0.0014	0.2124**	0.1493***	0.0061
L5.h_d_lnwlry	0.1026*	0.0753	-0.0255	-0.0190
L5.h_d_lndjglmd	0.0727	0.2187***	-0.0356	0.0176

Note

* ** *** represents the significance levels of 10%,5% and 1% respectively.

where, h_ represents the variable after eliminating the fixed effect by Helmert transform. L1, L2, L3, L4, L5 indicate lag first order to lag fifth order.

In the results shown in Table 15, when the explanatory variable is manufacturing MI, its own lag first-order to fifth-order have significant negative effects; The first, third and fifth-order lag of freight turnover has insignificant negative effect, and the second and fourth-order lag has significant negative effect; The number of logistics industry employees lag first-order, second-order has insignificant positive effect, lag third-order, fifth-order has significant positive effect, lag fourth-order has insignificant negative effect; The first, third and fifth-order lag of the density of the grade highway network has insignificant positive effect, the second-order lag has insignificant negative effect, and the fourth-order lag has significant positive effect, which is manifested as promotion–inhibition–promotion–significant promotion–promotion. When the explained variable is freight turnover, its own lag from first-order to fifth-order, which is manifested as inhibition–promotion–inhibition–inhibition–significant promotion; Manufacturing MI is characterized by inhibition–significant inhibition–inhibition–promotion–promotion; The number of logistics industry employees shows inhibition–significant inhibition–inhibition–promotion–promotion; The density of the grade highway network shows significant promotion–significant promotion–inhibition–promotion–significant promotion. When the explained variable is the number of logistics industry employees, the first to fifth order of its own lag is promotion–promotion–promotion–promotion–inhibition; Manufacturing MI shows promotion–significant promotion–promotion–promotion—inhibition; The freight turnover shows significant promotion–promotion–significant promotion–promotion—significant promotion; The density of the grade highway network shows significant inhibition–inhibition–significant inhibition–promotion–inhibition.

When the explained variable is the density of the grade highway network, its own lag from first order to fifth order is significant promotion–promotion–inhibition–significant inhibition–promotion; Manufacturing MI shows inhibition–inhibition–significant promotion–promotion–significant promotion; The freight turnover shows promotion–significant promotion–promotion—significant promotion–promotion; The number of logistics industry employees show inhibition–inhibition–promotion–significant inhibition–inhibition.

#### Stability of the model and Granger causality test

Set the optimal lag order as 5 to further verify the model’s stability of logistics industry and the manufacturing MI, and the Granger causality between variables, all characteristic roots (including real roots and virtual roots) are less than 1, therefore, the PVAR model is stable and passes the stability test. Then, Granger causality test with a lag of 5 periods was conducted for each variable [69], and the results shown in Table 16 were obtained.

**Table 16:** Granger causality test of logistics industry and the manufacturing MI.

Original hypothesis: the latter is not the Granger reason of the former			p-value	Conclusion
h_d_lngymi	-	h_d_lnzzl	0.129	rejected
h_d_lngymi	-	h_d_lnwlry	0.195	rejected
h_d_lngymi	-	h_d_lndjglmd	0.139	rejected
h_d_lnzzl	-	h_d_lngymi	0.200	rejected
h_d_lnzzl	-	h_d_lnwlry	0.010	supported
h_d_lnzzl	-	h_d_lndjglmd	0.000	supported
h_d_lnwlry	-	h_d_lngymi	0.036	supported
h_d_lnwlry	-	h_d_lnzzl	0.000	supported
h_d_lnwlry	-	h_d_lndjglmd	0.036	supported
h_d_lndjglmd	-	h_d_lngymi	0.011	supported
h_d_lndjglmd	-	h_d_lnzzl	0.054	supported
h_d_lndjglmd	-	h_d_lnwlry	0.175	rejected

According to Table 16, the freight turnover, the number of logistics industry employees and the density of the grade highway network are not Granger reasons for manufacturing MI, manufacturing MI is not the Granger reason for the freight turnover, the number of logistics industry employees and the density of the grade highway network are Granger reasons for the freight turnover, manufacturing MI, freight turnover and the density of the grade highway network are all Granger reasons for the number of logistics industry employees, manufacturing MI and freight turnover are Granger reasons for the density of the grade highway network, the number of logistics industry employees is not the Granger reason for the density of the grade highway network. It shows that the impact of the current logistics industry on the change rate of green total factor productivity of manufacturing industry is not obvious, or its early changes cannot effectively explain the change of green total factor productivity of manufacturing industry, while the change of green total factor productivity of manufacturing industry mainly affects the scale of logistics integrator and the level of logistics connection-key.

#### PVAR impulse response analysis

**Fig 3:**
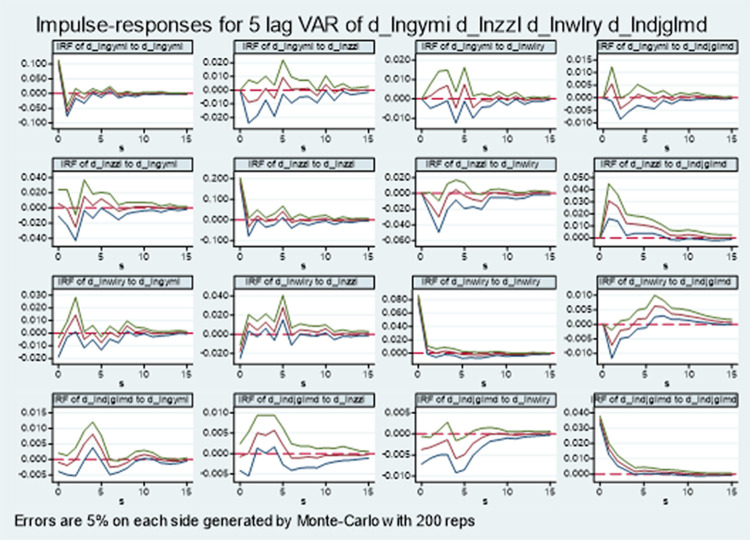
Impulse response diagram of logistics industry and manufacturing MI.

In order to fully describe the long-term dynamic impact effect between logistics industry and the change rate of green total factor productivity in manufacturing industry, the impulse response function is further analyzed, and the 95% confidence interval impulse response diagram of other endogenous variables is calculated 200 times by Monte Carlo random simulation, as shown in Fig 3. The horizontal axis represents the number of response periods, the vertical axis represents the magnitude of impulse response value, the upper and lower lines represent the confidence interval of 5% ~ 95%, and the middle line represents the estimation curve of impulse response function.

For a standard deviation shock from the manufacturing MI itself, it immediately produces a strong positive response to reach the peak, then quickly reduces and converges to 0 in the fluctuation, indicating that the manufacturing MI is highly dependent on itself. A standard deviation impact of freight turnover on manufacturing MI shows a positive response at the beginning, becomes negative in the first lag period, then reaches the peak in the second lag period, and then converges to 0 in the fluctuation. The impact of a standard deviation impact from the number of logistics industry employees on manufacturing MI makes it produce a peak negative response when it lags behind phase 1, and then converge to 0 in the fluctuation after it reaches the peak in lag phase 2. At first, a standard deviation impact from the density of the grade highway network on manufacturing MI was a weak negative effect, then rose to the peak in lag phase 4, then began to decline and converge to 0 in the fluctuation.

For freight turnover, a standard deviation impact from manufacturing MI starts to be negative in lag phase 1, then rises to be positive in lag phase 5, and converges to 0 in fluctuation. For a standard deviation impact from the freight turnover itself, it reaches a positive peak at the beginning, and then weakens rapidly and converges to 0. For a standard deviation impact of the number of logistics industry employees on freight turnover, it was a negative response at first, then increased, and reached a positive peak in the fifth period, then decreased and gradually converged to 0. For a standard deviation impact from the density of the grade highway network, the freight turnover showed a relatively high positive response in the early stage, and then a weak negative response and converged to 0 after phase 5.

For the number of logistics industry employees, the impact of manufacturing MI gradually increases from 0 to positive, then it is negative in the fourth lag period, reaches the peak in the fifth lag period, and then converges to 0 in positive and negative fluctuations. The impact of a standard deviation shock from freight turnover starts from 0 to negative, reaches the trough in the second lag period, then rises, and converges to 0 in positive and negative fluctuations. For a standard deviation impact from the number of logistics industry employees themselves, the number of logistics industry employees first reach the positive peak, and then quickly decline to 0. For a standard deviation impact from the density of the grade highway network, the number of logistics industry employees initially reacted negatively, then rose in fluctuation and converged to 0 in the seventh lag period.

For the density of the grade highway network, a standard deviation impact from manufacturing MI reached a positive peak in the lag phase, then decreased to the trough in the lag phase 2, and then began to fluctuate upward and converge to 0. For a standard deviation impact from freight turnover, it reaches the peak in the first lag period, and then converges to 0 in the fluctuation. A standard deviation impact of the number of logistics industry employees reached the trough in the first lag period, then rose continuously in the fluctuation, reached the peak in the sixth lag period, and converged gently to 0. For a standard deviation impact from the density of the grade highway network itself, it reaches the positive peak at the beginning, and then converges to 0.

#### Variance decomposition

The variance decomposition results of the interaction between logistics and manufacturing MI are shown in Table 17.

**Table 17:** Variance decomposition of logistics industry and manufacturing MI.

Variables	Phase	d_lngymi	d_lnzzl	d_lnwlry	d_lndjglmd
d_lngymi	1	1.000	0.000	0.000	0.000
d_lngymi	2	0.993	0.005	0.000	0.002
d_lngymi	3	0.987	0.008	0.002	0.003
d_lngymi	4	0.984	0.008	0.005	0.003
d_lngymi	5	0.980	0.010	0.006	0.003
d_lngymi	10	0.970	0.016	0.010	0.003
d_lngymi	15	0.968	0.018	0.011	0.004
d_lngymi	20	0.968	0.018	0.011	0.004
d_lngymi	25	0.968	0.018	0.011	0.004
d_lngymi	30	0.968	0.018	0.011	0.004
d_lnzzl	1	0.001	0.999	0.000	0.000
d_lnzzl	2	0.001	0.970	0.005	0.024
d_lnzzl	3	0.016	0.919	0.026	0.039
d_lnzzl	4	0.022	0.911	0.026	0.042
d_lnzzl	5	0.022	0.907	0.026	0.045
d_lnzzl	10	0.025	0.899	0.028	0.048
d_lnzzl	15	0.025	0.899	0.029	0.048
d_lnzzl	20	0.025	0.899	0.029	0.048
d_lnzzl	25	0.025	0.899	0.029	0.048
d_lnzzl	30	0.025	0.899	0.029	0.048
d_lnwlry	1	0.018	0.045	0.937	0.000
d_lnwlry	2	0.018	0.062	0.913	0.007
d_lnwlry	3	0.045	0.063	0.885	0.007
d_lnwlry	4	0.047	0.081	0.864	0.008
d_lnwlry	5	0.047	0.082	0.863	0.008
d_lnwlry	10	0.053	0.166	0.763	0.019
d_lnwlry	15	0.053	0.168	0.758	0.020
d_lnwlry	20	0.053	0.169	0.758	0.020
d_lnwlry	25	0.053	0.169	0.758	0.020
d_lnwlry	30	0.053	0.169	0.758	0.020
d_lndjglmd	1	0.001	0.000	0.011	0.988
d_lndjglmd	2	0.003	0.000	0.015	0.982
d_lndjglmd	3	0.003	0.015	0.017	0.965
d_lndjglmd	4	0.017	0.026	0.018	0.939
d_lndjglmd	5	0.051	0.042	0.035	0.873
d_lndjglmd	10	0.061	0.044	0.049	0.846
d_lndjglmd	15	0.063	0.045	0.049	0.844
d_lndjglmd	20	0.063	0.045	0.049	0.844
d_lndjglmd	25	0.063	0.045	0.049	0.844
d_lndjglmd	30	0.063	0.045	0.049	0.844

The results in Table 17 show that the variance contribution rate of manufacturing MI to itself tends to be stable from 100% lagging behind in phase 1 to 96.8% in phase 15, indicating that manufacturing MI is its largest contribution factor. The contribution of freight turnover, the number of logistics industry employees and the density of the grade highway network to manufacturing MI is relatively small at the beginning, but their contributions are gradually increasing, increasing to 1.8%, 1.1% and 0.4% respectively in the 10th lag period. It can be seen that from the perspective of logistics industry, the contribution to the change rate of green total factor productivity of manufacturing industry is the capacity of logistics base-nuclear from large to small, the scale of logistics integrator and the level of logistics connection-key.

The variance contribution rate of freight turnover to itself tends to be stable from 99.9% in phase 1 to 89.9% in phase 10, indicating that freight turnover is its biggest contribution factor. The contribution of manufacturing MI and the density of the grade highway network to freight turnover increased to 2.5% and 4.8% respectively in the 10th lag period, and the impact of the number of logistics industry employees on it increased to 2.9% in the 15th lag period. It can be seen that from the perspective of logistics industry, the contribution to the development of the capacity of logistics base-nuclear is in the order of the level of logistics connection-key, the scale of logistics integrator, and the change rate of green total factor productivity in manufacturing industry.

The variance contribution rate of the number of logistics industry employees to themselves tends to be stable from 91.3% in phase 1 to 75.8% in phase 15, indicating that the number of logistics industry employees are their biggest contribution factor. The contribution of manufacturing MI to the number of logistics industry employees reached 5.3% in the 10th period, the contribution of freight turnover to it reached 16.9% in the 20th period, the density of the grade highway network reached 2% in the 15th period. It shows that from the perspective of logistics industry, the contribution to the scale of logistics integrator from large to small is the capacity of logistics base-nuclear, the change rate of green total factor productivity of manufacturing industry and the level of logistics connection-key.

The variance contribution rate of the density of the grade highway network to itself tends to be stable from 98.8% in phase 1 to 84.4% in phase 15, indicating that the density of the grade highway network is its largest contribution factor. In the manufacturing industry, the impact of freight turnover and the density of the grade highway network on it increased to 6.3% and 4.5% in the 15th lag period, and the number of logistics industry employees reached 4.9% in the 10th lag period. It can be seen that from the perspective of logistics industry, the contribution to the level of logistics connection-key from large to small is the change rate of green total factor productivity of manufacturing industry, the scale of logistics integrator and the capacity of logistics base-nuclear.

In the long run, the impact of the change rate of green total factor productivity of manufacturing industry on other factors from large to small is the density of the grade highway network, the number of logistics industry employees and the freight turnover, that is, the level of logistics connection-key, the scale of logistics integrator and the capacity of logistics base-nuclear.

### Interaction between logistics industry and the efficiency of the two-industry-linkage from the perspective of integration field

#### Unit root test of variables

The relevant time series data are logarithmically processed, the processed data are the logarithm of the efficiency of two-industry-linkage (lnwgd), the logarithm of the freight turnover (lnzzl), the logarithm of the number of logistics industry employees (lnwlry), and the logarithm of the density of the grade highway network (lndjglmd). The unit root stationarity test is conducted for the variable series. According to the statistical characteristics of the data, HT and IPS were selected as the test standards [65]. The test results by using stata16 software are shown in Table 18.

**Table 18:** Description of unit root test results of variables.

Variables	Inspection method	Statistic	p-value	Conclusion
lnwgd	HT	0.5847	0.0739	stable
	IPS	-4.5352	0.0000	stable
lnzzl	HT	0.5169	0.0007	stable
	IPS	-1.3768	0.0843	stable
lnwlry	HT	0.5913	0.0998	stable
	IPS	-4.4015	0.0000	stable
lndjglmd	HT	0.7002	0.9231	unstable
	IPS	-1.8715	0.0306	stable

The test results show that some sequences are non-stationary at the significance level of 10%. Then, the first-order difference sequence was tested and the results shown in Table 19 were obtained.

**Table 19:** Description of unit root test results of variable first-order difference sequence.

Variables	Inspection method	Statistic	p-value	Conclusion
d_lnwgd	HT	-0.0680	0.0000	stable
	IPS	-11.7434	0.0000	stable
d_lnzzl	HT	-0.1739	0.0000	stable
	IPS	-9.3001	0.0000	stable
d_lnwlry	HT	-0.0154	0.0000	stable
	IPS	-11.6296	0.0000	stable
d_lndjglmd	HT	0.1008	0.0000	stable
	IPS	-9.7387	0.0000	stable

The test results show that at the significance level of 1%, the first-order difference sequences are stable, i.e. I(1), and there is a long-term stable proportional relationship between them. Some variables in the original data series are unstable. According to the unit root test of the above variables, all variables (lnwgd, lnzzl, lnwlry, lndjglmd) belong to first-order cointegration. According to the cointegration theory, the efficiency of the two-industry-linkage (lnwgd) and the development variables of the logistics industry (lnzzl, lnwlry, lndjglmd) constitute a long-term equilibrium relationship. The cointegration test is carried out to examine whether there is a long-term equilibrium cointegration relationship between the variables, the test results are shown in Table 20, most of the tests reject the original test without cointegration relationship, that is, it can be judged that there is cointegration relationship between variables.

**Table 20:** Cointegration test of logistics industry and the efficiency of two-industry-linkage.

		Statistic	p-value
Kao test	Modified Dickey-Fuller t	-20.8575	0.0801
	Dickey-Fuller t	-19.0564	0.0054
	Augmented Dickey-Fuller t	-11.9155	0.3913
	Unadjusted modified Dickey Fuller t	-24.2188	0.0903
	Unadjusted Dickey-Fuller t	-19.3999	0.0060
Pedroni test	Modified Phillips-Perron t	-0.1912	0.4242
	Phillips-Perron t	-11.3056	0.0000
	Augmented Dickey-Fuller t	-11.9484	0.0000
Westerlund test	Variance ratio	-1.7388	0.0410

#### Determine the optimal lag order

The PVAR model for logistics industry and the efficiency of two-industry-linkage was established, first determine the lag order, select it by using Lian Yujun’s stata16.0 software package pvar2, and determine the lag period according to the minimum criteria such as AIC and SC, the results are shown in Table 21.

**Table 21:** Lag test of PVAR model for logistics and the efficiency of the two-industry-linkage.

lag	AIC	BIC	HQIC
1	-5.6878	-4.50522	-5.22295
2	-5.76426	-4.37625	-5.2172
3	-5.70942	-4.09332	-5.07066
4	-5.6837	-3.81249	-4.94194
5	-7.26302*	-5.10407*	-6.40458*

It can be seen from Table 21 that the lag period of PVAR model for logistics industry and the efficiency of two-industry-linkage is 5. The PVAR(5) model was established by using stata16.0 software, and the estimation results are shown in Table 22.

**Table 22:** GMM estimation results of PVAR model for logistics industry and the efficiency of two-industry-linkage.

	h_d_lnwgd	h_d_lnzzl	h_d_lnwlry	h_d_lndjglmd
L.h_d_lnwgd	0.1414**	-0.2222	0.1460*	-0.0061
L.h_d_lnzzl	-0.0076	-0.1370	0.0557**	0.0073
L.h_d_lnwlry	-0.0620*	-0.0838	-0.0352	-0.0301
L.h_d_lndjglmd	0.0239	0.1718**	0.0297	0.1526***
L2.h_d_lnwgd	-0.0252	-0.0220	0.0644	0.0488
L2.h_d_lnzzl	0.0089	0.0539	0.0431*	0.0263**
L2.h_d_lnwlry	-0.0246	-0.3245**	0.0422	-0.0040
L2.h_d_lndjglmd	-0.1204***	0.6274***	-0.0729*	0.0687**
L3.h_d_lnwgd	-0.0436	0.2711*	-0.2398***	0.0420
L3.h_d_lnzzl	-0.0315*	0.0150	0.0780***	0.0257**
L3.h_d_lnwlry	0.0297	-0.0507	0.0655	-0.0088
L3.h_d_lndjglmd	0.0163	-0.0588	-0.0337	0.0212
L4.h_d_lnwgd	-0.1032**	-0.0360	-0.0671	0.0411
L4.h_d_lnzzl	0.0263	0.0151	0.0351	0.0326***
L4.h_d_lnwlry	-0.1592***	0.0494	0.0970***	-0.0759***
L4.h_d_lndjglmd	-0.0792**	0.1011	-0.0022	-0.0454
L5.h_d_lnwgd	-0.1042*	0.2068	-0.1076*	0.0464
L5.h_d_lnzzl	-0.0030	0.2335***	0.1390***	0.0235*
L5.h_d_lnwlry	0.0165	-0.0438	0.0283	-0.0366
L5.h_d_lndjglmd	0.0468	0.1625**	-0.0194	-0.0103

Note

* ** *** represents the significance levels of 10%,5% and 1% respectively.

where, h_ represents the variable after eliminating the fixed effect by Helmert transform. L1, L2, L3, L4, L5 indicate lag first order to lag fifth order.

In the results shown in Table 22, when the explained variable is the efficiency of two-industry-linkage, the first-order lag has a significant positive effect, the second-order lag, the third-order lag have an insignificant negative effect, the fourth-order and fifth-order lag have a significant negative effect, which is shown as significant promotion–inhibition–inhibition–significant inhibition–significant inhibition; The first and fifth order lag of freight turnover has insignificant negative effect, the second and fourth order lag have insignificant positive effect, and the third order lag has significant negative effect, which is manifested as inhibition–promotion–significant inhibition–promotion–inhibition; The first-order and fourth-order lag of the number of logistics industry employees has significant negative effect, the second-order lag has insignificant negative effect, the third-order lag and the fifth-order lag have insignificant positive effect, which is manifested as significant inhibition–inhibition–promotion–significant inhibition–promotion; The first-order and third-order lag of the density of the grade highway network has insignificant positive effect, the second-order lag and the fourth-order lag have significant negative effect, and the fifth-order lag has insignificant positive effect, which is shown as promotion–significant inhibition–promotion–significant inhibition–promotion. When the explained variable is freight turnover, its own lag from first order to fifth order is inhibition–promotion–promotion–promotion–significant promotion; The efficiency of two-industry-linkage shows inhibition–inhibition–significant promotion–inhibition–promotion; The number of logistics industry employees shows inhibition–significant inhibition–inhibition–promotion–inhibition; The density of the grade highway network shows significant promotion–significant promotion–inhibition–promotion–significant promotion. When the explained variable is the number of logistics industry employees, the first to fifth is inhibition–promotion–promotion–significant promotion–promotion; The efficiency of two-industry-linkage shows significant promotion–promotion–significant inhibition–inhibition–significant inhibition; The freight turnover is significant promotion–significant promotion–significant promotion–significant promotion–promotion; The density of the grade highway network shows promotion–significant inhibition–inhibition–inhibition–inhibition. When the explained variable is the density of the grade highway network, its own lag from first-order to fifth-order is significant promotion–significant promotion–promotion–inhibition–inhibition; The efficiency of two-industry-linkage shows inhibition–promotion–promotion–promotion–promotion; The freight turnover shows promotion–significant promotion–significant promotion–significant promotion–significant promotion; The number of logistics industry employees show inhibition–inhibition–inhibition–significant inhibition–inhibition.

#### Stability of the model and Granger causality test

Set the optimal lag order as 5 to further verify the model’s stability of logistics industry and the efficiency of the two-industry-linkage, and the Granger causality between variables, all characteristic roots (including real roots and virtual roots) are less than 1, therefore, the PVAR model is stable and passes the stability test. Then, Granger causality test with a lag of 5 periods was conducted for each variable [69], and the results shown in Table 23 were obtained.

**Table 23:** Granger causality test of logistics industry and the efficiency of two-industry-linkage.

Original hypothesis: the latter is not the Granger reason of the former			p-value	Conclusion
h_d_lnwgd	-	h_d_lnzzl	0.164	supported
h_d_lnwgd	-	h_d_lnwlry	0.001	rejected
h_d_lnwgd	-	h_d_lndjglmd	0.002	rejected
h_d_lnzzl	-	h_d_lnwgd	0.057	rejected
h_d_lnzzl	-	h_d_lnwlry	0.086	rejected
h_d_lnzzl	-	h_d_lndjglmd	0.000	rejected
h_d_lnwlry	-	h_d_lnwgd	0.001	rejected
h_d_lnwlry	-	h_d_lnzzl	0.000	rejected
h_d_lnwlry	-	h_d_lndjglmd	0.129	supported
h_d_lndjglmd	-	h_d_lnwgd	0.177	supported
h_d_lndjglmd	-	h_d_lnzzl	0.042	rejected
h_d_lndjglmd	-	h_d_lnwlry	0.019	rejected

According to Table 23, in the process of China’s overall economic development, the number of logistics industry employees and the density of the grade highway network are the Granger reasons for the efficiency of two-industry-linkage. The efficiency of two-industry-linkage, the number of logistics industry employees and the density of the grade highway network are the Granger reasons for the freight turnover. The efficiency of two-industry-linkage and the freight turnover are the Granger reasons for the number of logistics industry employees. The freight turnover, the number of logistics industry employees are Granger reasons for the density of the grade highway network. It shows that at present, the main impact on the efficiency of two-industry-linkage is the scale of logistics integrator and the level of logistics connection-key, the capability of logistics base-nuclear on it is not obvious, while the efficiency of two-industry-linkage mainly affects the capability of logistics base-nuclear and the scale of logistics integrator. Within the logistics industry, the interaction effect of the capability of logistics base-nuclear and the scale of logistics integrator on the level of logistics connection-key are obvious, the scale of logistics integrator and the level of logistics connection-key have an obvious impact on the capability of base-nuclear logistics, the capability of logistics base-nuclear has an obvious impact on the scale of logistics integrator.

#### PVAR impulse response analysis

**Fig 4:**
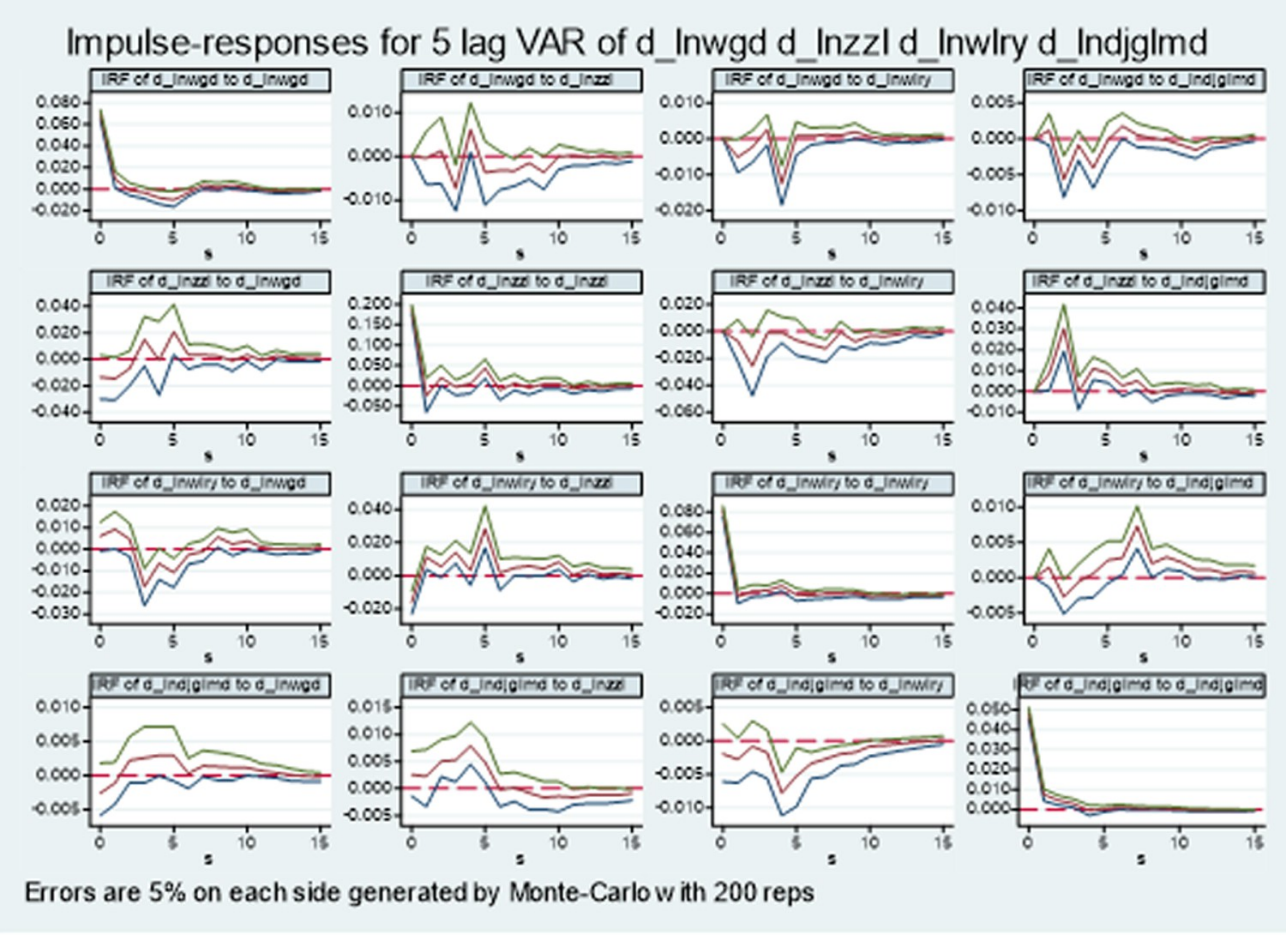
Impulse response diagram of logistics industry and the efficiency of two-industry-linkage.

In order to fully describe the long-term dynamic impact effect between logistics industry and the efficiency of two-industry-linkage, the impulse response function is further analyzed, and the 95% confidence interval impulse response diagram of other endogenous variables is calculated 200 times by Monte Carlo random simulation, as shown in Fig 4. The horizontal axis represents the number of response periods, the vertical axis represents the magnitude of impulse response value, the upper and lower lines represent the confidence interval of 5% ~ 95%, and the middle line represents the estimation curve of impulse response function.

For a standard deviation impact from the efficiency of two-industry-linkage itself, it immediately produces a strong positive response, reaches the peak, and then quickly reduces to 0, indicating that the coupling and coordinated development of the two industries is highly dependent on itself, that is, it needs the joint and coordinated development of high-quality manufacturing industry and high-quality logistics industry, but this inertia gradually weakens. For a standard deviation impact from the freight turnover, the efficiency of two-industry-linkage initially showed a negative response, became positive in the third lag period, and then reached the peak in the fifth lag period, and then most of them converged to 0. This shows that the increase of the freight turnover mainly depends on the strengthening of cooperation between the two industries, but in the process of the increase of the freight turnover, some invalid transportation distances and the increase of transportation and transshipment times are not conducive to the high-quality development of economy and even the high-quality development of logistics industry, which will also have some adverse effects on the coupling and coordinated development of the two industries. From the perspective of the capacity of logistics base-nuclear, when it is expanded, the capacity of logistics base-nuclear blindly exceeding the demand will cause a waste of resources, on the contrary, it is not conducive to the high-quality development of the logistics industry and the coupling and coordinated development of the two industries. The matching between the development of the capability of logistics base-nuclear and the efficiency of two-industry-linkage needs to go through a certain stage of practice and running in. When there is an error matching between the two industries, the capability of logistics base-nuclear will inhibit the coupling and coordinated development of the two industries. For a standard deviation impact from the number of logistics industry employees, the efficiency of two-industry-linkage initially shows a positive response, then reaches the peak in lag phase 1, then decreases rapidly, reaches the trough in lag phase 3, and then rises in fluctuation, after lag phase 7, it continues to show a weak positive effect and converges to 0, which shows that, the excessive increase of the number of logistics industry employees will cause a waste of market capacity. The emergence or expansion of logistics integrator is more to meet the needs of the market. It takes a period of market running in to show a positive promotion for the coupling and coordinated development of the two industries. For a standard deviation impact from the density of the grade highway network, the efficiency of two-industry-linkage shows a negative response at first, then rises to positive in the lag phase 1, reaches the peak in the lag phase 5, becomes 0 in the lag phase 6, and converges to 0. The increase of the density of the grade highway network and even the improvement of the level of logistics connection-key have driven the respective development of the two industries and even the linkage development of the two industries. With the gradual improvement of the high-quality development of the two industries in the later stage, the optimization of the linkage system of the two industries has been gradually realized.

For the freight turnover, a standard deviation impact from the efficiency of two-industry-linkage makes it fluctuate up and down in the lag period 1 ~ 10, which also confirms the above-mentioned that the coupling and coordinated development of the two industries, the generation of freight turnover and even the capacity of logistics base-nuclear need some practice and running in, and it is necessary to avoid error matching as much as possible. For a standard deviation impact from the freight turnover itself, it also reached a positive peak at the beginning, and then weakened rapidly and converged to 0. This also shows that the rapid expansion of the capacity of logistics base-nuclear depends on itself, but the long-term development also needs the influence of the outside world. For a standard deviation impact from the number of logistics industry employees, the freight turnover was negative at first, then positive when it lagged behind the first period, reached the peak in the fifth period, then decreased to 0 and converged to 0, the overall performance was positive, which also shows the important role played by the number of logistics industry employees in completing freight turnover. At the same time, it also further explains the impact of the expansion of the scale of logistics integrator on the further demand of the capability of logistics base-nuclear. For a standard deviation impact from the density of the grade highway network, the freight turnover initially showed a positive response, reached the peak in the fourth lag period, and then began to be negative and gradually converged to 0 after the fifth lag period. The reason is that when the density of the grade highway network increases, the freight turnover is more convenient, but it may also focus on surrounding businesses for a period of time, thus shortening the transportation distance. The impact of logistics connection-key on the capability of logistics base-nuclear is also positive in an appropriate range, blindly developing logistics connection-key is not necessarily conducive to the expansion of the capability of logistics base-nuclear. For example, the excessive increase of the density of the grade highway network will promote more incentive competition between logistics base-nuclear.

For the number of logistics industry employees, a standard deviation impact from the efficiency of two-industry-linkage is mostly negative, and it starts to be a weak positive response in the lag phase 5, and converges to 0. The increase of the number of logistics industry employees is reflected in the increase of investment in the high-quality development of the logistics industry, which reduces the high-quality efficiency of logistics and is not conducive to the development of the efficiency of two-industry-linkage, the coupling and coordinated development of the two industries depends more on low investment and high return. When the economy develops to a certain extent, the increase of the number and scale of logistics integrator is not necessarily beneficial to the coupling and coordinated development of the two industries. For a standard deviation impact from freight turnover, most number of logistics industry employees react negatively. For a standard deviation impact from the number of logistics industry employees themselves, their response reached the peak at the beginning, and then gradually began to converge to 0, which also shows that the number of logistics industry employees are more dependent on themselves, and the logistics integrator is more dependent on their own ability and even resources. A standard deviation impact from the density of the grade highway network on the number of logistics industry employees is mostly negative and fluctuates violently, it reaches the trough in the fourth lag period and gradually begins to converge upward to 0.

For the density of the grade highway network, a standard deviation impact from the efficiency of two-industry-linkage fluctuates greatly, it reaches the trough when it behind the third stage, and then fluctuates between positive and negative, which also proves that in the process of the linkage development of the two industries, the two need more wear and tear, and the increase of the density of the grade highway network, to some extent, it is conducive to the logistics integrator to improve its service level and better realize the linkage between the two industries. However, the excessive increase of the density of the grade highway network increases the investment in the logistics industry, which is not conducive to the high-quality development of the logistics industry, and then not conducive to the coupling and coordinated development of the two industries.

The development of the-two-industry linkage under the background of high-quality development poses a higher challenge to the development of logistics connection-key. For a standard deviation impact from the freight turnover, the response of the highway network density is positive, and reaches the peak in the second lag period, and then converges to 0 in the fluctuation. The increase of the freight turnover is a main reason for the increase of the highway network density. This also shows that the increase of the capacity of logistics base-nuclear is a main reason for the further development of logistics connection-key. For a standard deviation impact from the number of logistics industry employees, the response of the density of the grade highway network is initially a weak positive response, then a negative response, and begins to rise slowly. It reaches the peak in the seventh lag period, and then decreases in fluctuation and converges to 0. The improvement of the capacity of logistics integrator will also be accompanied by the improvement of the level of logistics connection-key. A standard deviation impact from the density of the grade highway network itself is positive at the beginning, and then converges to 0 rapidly, which also shows that the density of the grade highway network needs to rely more on the development of external factors.

#### Variance decomposition

The variance decomposition results of the interaction between logistics industry and the efficiency of two-industry-linkage are shown in Table 24.

**Table 24:** Variance decomposition of logistics industry and the efficiency of two-industry-linkage.

Variables	Phase	lnwgd	lnzzl	lnqc	lnlw
lnwgd	1	1.000	0.000	0.000	0.000
lnwgd	2	0.994	0.000	0.005	0.000
lnwgd	3	0.987	0.000	0.006	0.007
lnwgd	4	0.975	0.011	0.007	0.007
lnwgd	5	0.937	0.017	0.036	0.009
lnwgd	10	0.929	0.026	0.036	0.010
lnwgd	15	0.928	0.026	0.036	0.010
lnwgd	20	0.928	0.026	0.036	0.010
lnwgd	25	0.928	0.026	0.036	0.010
lnwgd	30	0.928	0.026	0.036	0.010
lnzzl	1	0.005	0.995	0.000	0.000
lnzzl	2	0.011	0.986	0.001	0.002
lnzzl	3	0.011	0.945	0.018	0.025
lnzzl	4	0.017	0.940	0.018	0.025
lnzzl	5	0.017	0.937	0.018	0.028
lnzzl	10	0.027	0.919	0.026	0.029
lnzzl	15	0.027	0.918	0.026	0.029
lnzzl	20	0.027	0.918	0.026	0.029
lnzzl	25	0.027	0.918	0.026	0.029
lnzzl	30	0.027	0.918	0.026	0.029
lnwlry	1	0.005	0.038	0.957	0.000
lnwlry	2	0.017	0.054	0.929	0.000
lnwlry	3	0.020	0.058	0.921	0.001
lnwlry	4	0.058	0.079	0.862	0.001
lnwlry	5	0.062	0.080	0.857	0.001
lnwlry	10	0.073	0.168	0.749	0.010
lnwlry	15	0.073	0.175	0.741	0.011
lnwlry	20	0.073	0.175	0.741	0.011
lnwlry	25	0.073	0.175	0.740	0.011
lnwlry	30	0.073	0.175	0.740	0.011
lndjglmd	1	0.003	0.003	0.002	0.993
lndjglmd	2	0.003	0.005	0.005	0.987
lndjglmd	3	0.005	0.015	0.005	0.975
lndjglmd	4	0.008	0.026	0.006	0.961
lndjglmd	5	0.011	0.048	0.028	0.913
lndjglmd	10	0.015	0.056	0.046	0.882
lndjglmd	15	0.016	0.059	0.047	0.878
lndjglmd	20	0.016	0.060	0.047	0.877
lndjglmd	25	0.016	0.060	0.047	0.877
lndjglmd	30	0.016	0.060	0.047	0.877

The results in Table 24 show that the contribution rate of the efficiency of two-industry-linkage to their own variance tends to be stable from 100% in the first period to 92.8% in the 15th period, indicating that the efficiency of two-industry-linkage is its biggest contribution factor. The contribution of freight turnover, the number of logistics industry employees and the density of the grade highway network to the efficiency of the two-industry-linkage is relatively small at the beginning, but the contributions of the three are gradually increasing. The freight turnover increased to 2.6% in the 10th lag period, the number of logistics industry employees increased to 3.6% in the 5th lag period, and the density of the grade highway network increased to 1% in the 10th lag period. It can be seen from the perspective of the logistics industry, from large to small, the contributions to the coupling and coordinated development of the two industries are the scale of logistics integrator, the capacity of logistics base-nuclear and the level of logistics connection-key, and these contributions are increasing day by day.

The variance contribution rate of freight turnover to itself tends to be stable from 99.5% in the first period to 91.8% in the 15th period, indicating that freight turnover is its biggest contribution factor. From the perspective of logistics industry, the contribution of the efficiency of two-industry-linkage, the number of logistics industry employees and the density of the grade highway network to the development of freight turnover increased to 2.7%, 2.6% and 2.9% respectively in the 10th period. It can be seen that from the perspective of logistics industry, the contribution to the development of the capacity of logistics base-nuclear is the level of logistics connection-key, the efficiency of two-industry-linkage and the scale of logistics integrator.

The variance contribution rate of the number of logistics industry employees to themselves tends to be stable from 95.7% in the first period to 74% in the 25th period, indicating that the number of logistics industry employees are their biggest contribution factor. The contribution of the efficiency of two-industry-linkage to the number of logistics industry employees has increased to 7.3% in the 10th lag period, and the freight turnover and the density of the grade highway network have increased to 17.5% and 1.1% respectively in the 15th lag period. It can be seen that from the perspective of the logistics industry, the contribution to the scale of the logistics integrator from large to small is the capacity of logistics base-nuclear, the efficiency of two-industry-linkage and the level of logistics connection-key.

The variance contribution rate of the density of the grade highway network to itself tends to be stable from 99.3% in phase 1 to 87.7% in phase 20, indicating that the density of the grade highway network is its largest contribution factor. The contribution of the coupling and coordinated development of the two industries and the number of logistics industry employees personnel to the density of the grade highway network reached 1.6% and 4.7% respectively in the 15th lag period, and the freight turnover reached 6% in the 20th lag period, indicating that the contribution to the development of the level of logistics connection-key from large to small is the capacity of logistics base-nuclear, the scale of logistics integrator and the efficiency of the two-industry-linkage.

In the long run, the impact of the efficiency of two-industry-linkage on other factors, from large to small, is the number of logistics industry employees, freight turnover, the density of the grade highway network, that is, the scale of logistics integrator, the capacity of logistics base-nuclear and the level of logistics connection-key. The influence of freight turnover on other factors from large to small is the number of logistics industry employees, the density of the grade highway network and the efficiency of two-industry-linkage, that is, the influence of the capacity of logistics base-nuclear on it from large to small is the scale of logistics integrator, the level of logistics connection-key and the efficiency of two-industry-linkage. The influence of the number of logistics industry employees on other factors from large to small is the density of the grade highway network, the efficiency of two-industry-linkage and the freight turnover, that is, the influence of the scale of logistics integrator on it from large to small is the level of logistics connection-key, the efficiency of two-industry-linkage and the capacity of logistics base-nuclear. The influence of the density of the grade highway network on other factors from large to small is the freight turnover, the number of logistics industry employees and the efficiency of two-industry-linkage, that is, the influence of the level of logistics connection-key on it from large to small is the capacity of logistics base-nuclear, the scale of logistics integrator and the efficiency of two-industry-linkage.

## Conclusion

At present, the impact of high-quality development of logistics industry on high-quality development of manufacturing industry is not obvious, manufacturing industry mainly affects the scale of logistics integrator and the level of logistics connection-key. The influence of the comprehensive technical efficiency of manufacturing on other factors from large to small is the scale of logistics integrator, the capacity of logistics base-nuclear and the level of logistics connection-key. The influence of the change of green total factor productivity of manufacturing industry on other factors from large to small is the level of logistics connection-key, the scale of logistics integrator and the capacity of logistics base-nuclear. The impact of the efficiency of two-industry-linkage on other factors from large to small is the scale of logistics integrator, the capacity of logistics base-nuclear and the level of logistics connection-key. Within the logistics industry, the interaction effects of the capability of logistics base-nuclear, the scale of logistics integrator and the level of logistics connection-key are obvious. The contribution of the high-quality development of logistics industry to the high-quality development of manufacturing industry from large to small are the capacity of logistics base-nuclear, the scale of logistics integrator and the level of logistics connection-key. From large to small, the contributions to the coupling and coordinated development of the two industries are the scale of logistics integrator, the capacity of logistics base-nuclear and the level of logistics connection-key, and these contributions are increasing day by day. The high-quality development and improvement of manufacturing industry needs more efficient logistics operation, the coupling and coordinated development of the two industries requires the joint and coordinated development of high-quality manufacturing industry and high-quality logistics industry, but this inertia gradually weakens. The development of logistics industry and manufacturing industry need to go through a certain stage of practice and running in, when there is an error matching between the two industries, the logistics industry will inhibit the coupling and coordinated development of the two industries. At present, in order to improve the linkage level of the two industries, the first choice is to improve the scale of logistics integrator, followed by the capacity of logistics base-nuclear and the level of logistics connection-key. However, it should also be noted that when the economy develops to a certain extent, the overall impact of the expansion of the scale of the logistics system on the coupling and coordinated development of the two industries is not necessarily beneficial. Blindly exceeding the demand for logistics investment will cause a waste of resources, which is not conducive to the high-quality development of the logistics industry and the coupling and coordinated development of the two industries.

The high-quality development of China’s logistics industry and manufacturing industry is mainly caused by the change of scale, but there is no obvious change in technical efficiency, which also provides a way for the high-quality development of the two-industry-linkage in the future. The high-quality development of China’s logistics industry and manufacturing industry is close on the whole, and the development trend is consistent. However, the level of the two-industry-linkage mostly belongs to the situation of low-level mutual restriction, which has not yet reached a high level of mutual promotion, resulting in the overall coupling coordination degree basically in a state of barely coordination. The main reason for this situation is that the development level of the comprehensive technical efficiency of the logistics industry and the manufacturing industry is inconsistent, and once the logistics industry improves its green total factor productivity, it may reduce some efficiency of the manufacturing industry and have some adverse effects on the benefit growth of the manufacturing industry. However, in the long run, the change of the logistics basic-nuclear capacity, the logistics integrator scale and logistics connection-key level will have a positive impact on the change of green total factor productivity in manufacturing industry. High-quality development is the inevitable trend of China’s economic development, and even the direction of the world’s future economic development. This long-term development needs the support of the corresponding policy environment, including the support for innovation, the restriction of green development, the promotion of coordinated and shared development, the improvement of the open development policy and the improvement of relevant systems, it should continue to deepen the reform of "decentralization, management and service", eliminate the institutional disadvantages restricting the market mechanism, implement the fair competition supervision mechanism and standardize the government behavior. High-quality development must attach importance to regional coordinated development, instead of simply requiring all regions to reach the same level of economic and social development, it should recognize the objective differences, formulate logistics development strategies according to local conditions through improving regional strategic planning and other mechanisms, so as to promote high-quality development and better promote the development of developed and underdeveloped regions, realize the common development of the Eastern, Central, Western and Northeast regions, and promote regional cooperation and mutual assistance and interregional interest compensation. At the same time, according to the development level of the two-industry-linkage in different regions, enterprises also need to choose the appropriate two-industry-linkage mode according to their own situation.

The coupling coordination model was used to theoretically analyze and empirically test the coupling and coordinated development level of high-quality development of logistics industry and manufacturing industry, based on the perspective of integration field theory, it takes the three basic synthetic fields of logistics integrator logistics base-nuclear and logistics connection-key as the analysis dimension, to introduce PVAR model into the two-industry-linkage for in-depth analysis and empirical test, it is novel and effective.

In addition, the manufacturing industry can be subdivided into different industries. In view of this, we will also try to analyze the impact of logistics industry on different manufacturing industries in the future, the coupling and coordinated development of logistics industry and different manufacturing industries, and on the basis of the existing model, we will further discuss the role of logistics integrator, logistics base-nuclear and logistics connection-key in different industries, conduct more empirical tests on the integration field theory.
